# Bone marrow-derived epithelial cells and hair follicle stem cells contribute to development of chronic cutaneous neoplasms

**DOI:** 10.1038/s41467-018-07688-8

**Published:** 2018-12-13

**Authors:** Heuijoon Park, Sonali Lad, Kelsey Boland, Kelly Johnson, Nyssa Readio, Guangchun Jin, Samuel Asfaha, Kelly S. Patterson, Ashok Singh, Xiangdong Yang, Douglas Londono, Anupama Singh, Carol Trempus, Derek Gordon, Timothy C. Wang, Rebecca J. Morris

**Affiliations:** 1 Department of Pathology and Cell Biology, Columbia University, New York, 10032 NY USA; 2 Department of Dermatology, Columbia University, New York, 10032 NY USA; 3 Division of Digestive and Liver Diseases, Department of Medicine and Irving Cancer Center, Columbia University, New York, 10032 NY USA; 4 The Hormel Institute, University of Minnesota, Austin, 55912 MN USA; 5 Department of Genetics, Rutgers, The State University of New Jersey, Piscataway, 08854-8082 NJ USA; 6 Matrix Biology Group, Immunity, Inflammation, and Disease Laboratory, National Institute of Environmental Health Sciences, Research Triangle Park, 27709 NC USA

## Abstract

We used allogeneic bone marrow transplantation (BMT) and a mouse multistage cutaneous carcinogenesis model to probe recruitment of bone marrow-derived epithelial cells (BMDECs) in skin tumors initiated with the carcinogen, dimethylbenz[*a*]anthracene (DMBA), and promoted with 12-*O*-tetradecanolyphorbol-13-acetate (TPA). BMDECs clustered in the lesional epithelium, expressed cytokeratins, proliferated, and stratified. We detected cytokeratin induction in plastic-adherent bone marrow cells (BMCs) cultured in the presence of filter-separated keratinocytes (KCs) and bone morphogenetic protein 5 (BMP5). Lineage-depleted BMCs migrated towards High Mobility Group Box 1 (HMGB1) protein and epidermal KCs in ex vivo invasion assays. Naive female mice receiving BMTs from DMBA-treated donors developed benign and malignant lesions after TPA promotion alone. We conclude that BMDECs contribute to the development of papillomas and dysplasia, demonstrating a systemic contribution to these lesions. Furthermore, carcinogen-exposed BMCs can initiate benign and malignant lesions upon tumor promotion. Ultimately, these findings may suggest targets for treatment of non-melanoma skin cancers.

## Abstract (web summary)

Bone marrow-derived epithelial cells can be recruited to sites of chronic inflammation. Here, the authors using allogenic bone marrow transplantation in a multistage murine cutaneous carcinogenesis model show that bone marrow-derived epithelial cells and hair follicle stem cells are recruited to cutaneous neoplasms during tumor promotion of carcinogen-exposed skin and bone marrow.

## Introduction

Stem cells have shown great promise for therapeutic and regenerative medicine. However, accumulating evidence has suggested a strong link between tissue stem cells and the origin of cancer. Many tumors have been proposed to have a heterogeneous cellular origin. We and others have demonstrated a role for keratin 15 (K15)-expressing hair follicle (HF) progenitor cells in skin tumorigenesis and have shown a contribution by both K15-positive and negative progenitors in papilloma development^1–3^. However, the source of the K15-negative cells in papillomas remains unknown. Possible sources include other epidermal stem/progenitor cells or stem cells from tissues such as the bone marrow (BM). We report here significant recruitment of BMCs to cutaneous epithelium in the contexts of tumor initiation and promotion.

Recent studies have implicated BMCs as a cellular source for tissue regeneration. In vitro co-culturing with bone morphogenetic protein 4 (BMP4) demonstrated that both hematopoietic stem cell (HSC) and mesenchymal stem cell (MSC) populations from the BM contributed to wound healing of several organs independently of cell fusion^4,5^. In vivo studies revealed that BMCs migrated preferentially to the site of injury and contributed to tissue regeneration^6–8^. Studies in skin have shown the plasticity of bone marrow-derived cells (BMDCs) and their contribution to epidermis during acute skin wound healing^9–13^. Multipotential plasticity of BMCs in the skin has been demonstrated by an in vivo grafting assay with genetically labeled (i.e., EGFP-expressing) BMCs^12^. Furthermore, acute cutaneous wound healing following allogeneic BMT revealed that genetically labeled or Y chromosome-positive BMDCs engrafted within the epidermal germinal layer at the site of the injury, and that a subset of BMDCs directly differentiated into cytokeratin and Ki67-expressing cells without cell fusion^9–11^. Recently, a subpopulation of platelet-derived growth factor receptor (PDGFR)-alpha-positive, non-hematopoietic BMDCs cells were found to engraft to skin following acute injury^13^. Despite these provocative observations, the number of recruited BMCs was quite small.

Several clinical observations also support the hypothesis that exogenous cellular sources contribute to cancer development. Detection of X and Y chromosomes by fluorescence in situ hybridization (FISH) has been a useful approach for identifying the donor origin of tumors in gender-mismatched allogeneic BM or organ transplanted recipients. Engraftment of donor-derived cells has been detected in epithelia of several cancers including glioblastoma, oral squamous cell carcinoma, lung cancer, gastrointestinal cancer, and skin carcinoma^14–17^. Studies with *Helicobacter* infection gastric cancer models showed that keratin-positive BMDCs were identified within the neoplastic gastric glands of infected mice^18,19^. These studies suggested that BMDCs might contribute to the development of chronic inflammation-mediated gastric cancer. However, with the exception of these latter two studies, the previous tumor models have not addressed severe chronic inflammation associated with epithelial hyperplasia characteristic of skin cancer or chronic non-healing cutaneous ulcers. Moreover, the clinical significance of BMC recruitment in the foregoing studies could not be determined due to low incidence of recruited cells and non-quantitative data.

Therefore, we addressed the contribution of BMDCs during skin carcinogenesis in the presence of chronic inflammation and epidermal hyperplasia. Here, we report that BMCs became keratin-immunoreactive in vitro in the absence of cellular contact or fusion. In vivo, chronic TPA treatment of mice recruited more clusters of BMDCs in hyperplastic epidermis than did acute TPA treatment alone. Significant numbers of proliferating BMDECs were detected in both chemically induced papillomas and ulcer-associated dysplasia. In ulcer-associated dysplasia, contribution of both BMDECs and progeny of K15-positive bulge stem cells was observed. Moreover, transplantation of BMCs from DMBA-exposed mice could initiate squamous skin lesions in naive recipients upon TPA promotion. We conclude that large numbers of BMDECs are recruited to a subset of cutaneous papillomas and dysplastic ulcers and reflect a previously unrecognized systemic contribution to these lesions.

Ultimately, these findings may contribute to the identification of potential therapeutic targets for the treatment of non-melanoma skin cancer as well as other cancers and may provide a novel source of progenitor cells for regenerative medicine.

## Results

### BMC/KC co-culture induced cytokeratin expression in BMCs

**Fig. 1:**
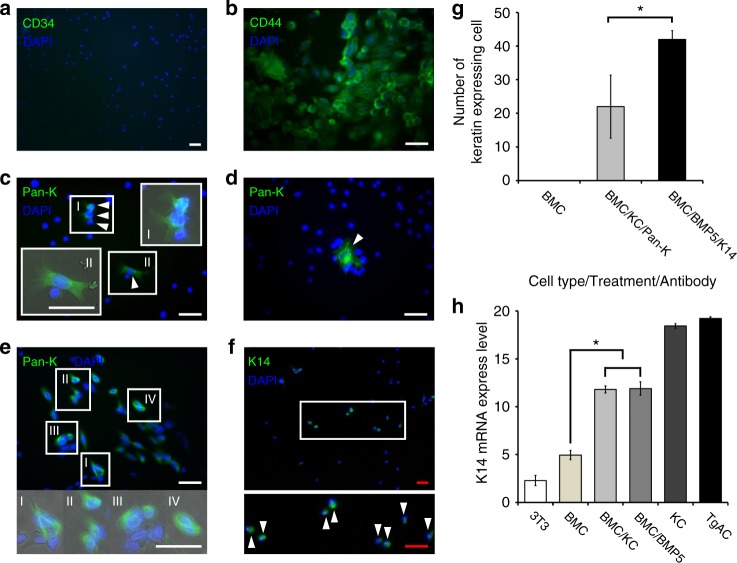
CD34^−^, CD44^+^ BMCs express keratin after BMC/KC co-culture and BMP5 treatment. **a**, **b** All adherent BMCs are CD34-negative and CD44-positive. **c**, **e** A sub set of adherent BMCs are pan-keratin-immunoreactive (arrowheads) after 7days of BMC/KC co-culture (keratin-positive BMCs in white boxes are magnified and merged with phase image). **d** Pan-keratin-immunoreactive BMC (arrowhead) identified 10 days after BMP5 treatment. **f** K14-immunoreactive cells (arrowheads) 10 days after BMP5 treatment (white box area is magnified). **g** Histogram of number of keratin-expressing BMCs; BMCs without treatment, BMC/KC co-culture (pan-keratin-positive BMCs, gray bar) and BMP5 treatment (K14-positive BMCs, black bar), pan-keratin- and K14-immunoreactive BMCs are detected in KC co-cultured and BMP5-treated BMCs, but no keratin-positive cells are detected in treatment controls (*n* = 3, 3 different culture groups, 3 male and 3 female mice in each group; *P* < 0.017 as determined by Student’s *t*-test, mean ± s.d.) **h** Q-RT-PCR results show the relative level of K14 expression detected from positive (primary KCs and Tg.AC, a KC cancer cell-line) and negative (Swiss mouse 3T3 cell-line and primary BMCs) control groups, and experimental (BMC/KC co-culture and BMP5 treatment) groups. The expression levels were normalized to GAPDH expression levels, and expression mean values were converted into fold-change gene expression. Final values were normalized with log_2_ transformation. (*n* = 3, *P* < 4.81 × 10E−12 as determined by Fisher’s ANOVA method, mean ± s.d.). *White scale bar, 50 µm; Red scale bar, 200 µm

To demonstrate the plasticity of BMCs, BMCs were co-cultured with primary KCs followed by identification of KC markers. Whole BMCs were harvested from the femurs and tibiae of male C57BL/6 mice, and plastic-adherent BMCs were co-cultured with 1-week-old primary mouse epidermal KCs separated by an impassable filter (Supplementary Figure 1a) in the presence of mouse MSC culture medium (MesenCult). Immunostaining confirmed that all plastic-adherent BMCs were CD34^−^, CD44^+^ (Fig. 1a, b). One week after co-culture, keratin expression was detected in the BMCs using a pan-keratin antibody. Tg.AC cells (a KC cancer cell-line developed from Tg.AC mice^20^), Swiss mouse 3T3 cells, and plastic-adherent BMCs without treatment were used as controls (Fig. 1c, e, Supplementary Figure 2). Pan-keratin immunoreactive BMCs were counted from the entire surface of the culture dishes, based on DAPI-positive nuclei and keratin immunoreactive cytoplasm (Fig. 1c, e, g). Initially, few keratin-positive BMCs were detected in the cultures, and no significant cell size and morphological differences were apparent between keratin-positive and negative BMCs. In addition, there was considerable variability in the number of keratin-expressing cells among different co-cultured cells. At later intervals, keratin 14 (K14) expression was detected from co-cultured BMC samples (Fig. 1h). Pan-keratin-immunoreactive and K14-immunoreactive cells were not detected in non-co-cultured BMC control groups. These experiments demonstrate that exposure of BMCs to a KC-derived microenvironment is able to induce keratin expression in a subset of the BMCs in the absence of cell contact.

### BMP5 induced keratin expression in plastic-adherent BMCs

We recently demonstrated that BMP5 was expressed in the epidermis and HF of normal adult mice, and that BMP5 added to KC cultures increases stem cell proliferation^21^. BMP5 expression was verified in mouse hyperplastic and dysplastic epidermis and epithelium of papilloma (Supplementary Figure 3). Therefore, we examined the effects of BMP5 on the keratinization of BMCs. BMP5 (50 ng/ml) was added to the BMC culture three times for 10 days following the first medium change (Supplementary Figure 1b). Following BMP5 treatment, pan-keratin-immunoreactive and K14-immunoreactive BMCs were detected in the cell cultures (Fig. 1d, f, Supplementary Figure 4). The number of K14-immunoreactive BMCs was counted using the same strategy as described above. The total number of K14-positive BMCs increased, but there was no significant difference in the level of K14 gene expression in BMP5-treated cells (Fig. 1g, h). Although K14 was most specific to the basal epithelium of skin, BMP5 treatment of BMC cultures resulted in more K14-positive BMCs than pan-keratin-positive BMCs following BMC/KC co-culture (Fig. 1g) and induced a more consistent number of keratin-expressing BMC than did KC co-culture (Fig. 1g). Similar to the KC co-cultures, we did not detect any significant changes in cell size or morphology. Thus, BMP5 treatment induced K14 expression in a subpopulation of BMCs in a consistent manner in different treatment groups, without morphological change.

### TPA-induced chronic inflammation leads to BMDC recruitment

**Fig. 2:**
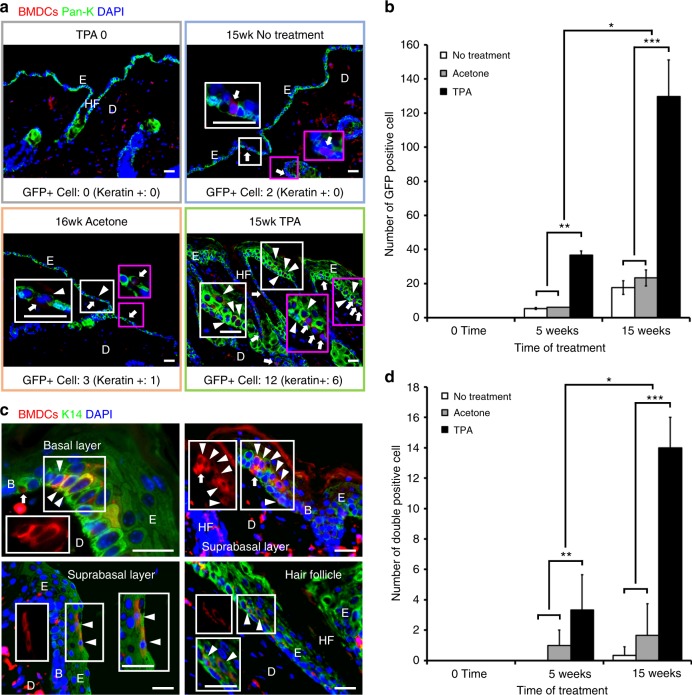
Long-term TPA treatment increases BMDCs in the epidermis of the skin. **a** BMDCs were detected in the epidermis of the skin with different treatment groups (before TPA treatment, no treatment, acetone-treated, and TPA-treated) and specific areas in white and magenta boxes are magnified. Arrows indicate keratin-negative BMDCs and arrowheads indicate keratin-positive BMDCs. **b** The number of GFP-positive cells in the epidermis are compared among three different treatment groups (no treatment, acetone-, and TPA-treated) at three different time points. The treatment types (no treatment, acetone, and TPA) are different across all weeks (*P* = 5.35 × 10E−11). The average time effects are different across all treatment levels (*P* = 1.32 × 10E−11). Additionally, the treatment type and time interact in a non-additive fashion (*P* = 6.6 × 10E−10). (*n* = 3, over all *P* value = 5.72 × 10E−13 as determined by the *F*-statistic, mean ± s.d.). **c** Keratin-positive BMDCs were observed in the basal layer, the suprabasal layer, and hair follicle. Areas in white boxes are magnified or shown in single color. Arrows indicate keratin negative BMDCs and arrowheads indicate keratin-positive BMDCs. **d** The number of GFP/Keratin-positive (double positive) BMDCs in the epidermis are compared among three different treatment groups (no treatment, acetone-, and TPA-treated) at three different time points. The number of double positive cells are increased in long-term TPA-treated groups. Both treatment type (acetone and TPA) and time (in weeks) have significant effects. This implies that the means of treatment types (no treatment, acetone, and TPA) have different effects across all weeks (*P* = 3.87 × 10E−08). The average time effects are different across all treatment types (*P* = 1.95 × 10E−07). Finally, there is a significant interaction term; this means that treatment type and time interact in a non-additive fashion (*P* = 1.53 × 10E−07). (*n* = 3, overall *P* value = 1.22 × 10E−09 as determined by the *F*-statistic, mean ± s.d.). *Epidermis (E), Dermis (D), Basal layer (B), Hair follicle (HF) **All counted cells are DAPI positive ***White scale bar, 50 µm

To demonstrate recruitment of BMDCs to chronic inflammatory sites such as hyperplastic epidermis or papillomas, we used a two-stage carcinogenesis model in C57BL/6 female mice that had undergone BMT with genetically marked (EGFP) whole BMCs from male donors. A single, subtumorigenic dose of DMBA (200 nmol in 200 µl of acetone) was applied to the dorsal skin of BMT recipients prior to BMT. Five weeks after gender-mismatched BMT, TPA (17 nmol in 200 µl of acetone) was applied three times per week to the dorsal skin of BMT recipients (DMBA ► BMT ► TPA, Supplementary Figure 5). In preliminary studies, KCs were harvested from TPA- and acetone- (vehicle control) treated mice, and a greater number of GFP-positive cells were observed in TPA-treated versus vehicle-treated control mice at both 4 and 9 weeks (Supplementary Figure 5a, Supplementary Figure 6). To extend these observations, we compared three different treatment groups (no treatment, acetone-, and TPA-treated), and skin tissues were collected at different time points (0, 5, and 15+ weeks). After immunostaining with GFP and pan-keratin antibodies, the number of GFP- and DAPI-positive BMDCs in the epidermis was counted. We did not detect any BMDCs before treatment (5 weeks post-BMT) (Fig. 2a, b). Following a short course (i.e., 5 weeks) of treatment, some solitary BMDCs were identified in the TPA-treated, but not in the no treatment or acetone-treated groups (Fig. 2a, b). Following long-term treatment, however, the number of BMDCs in TPA-induced hyperplastic skin was increased three-fold compared to skin from mice treated with TPA for 5 weeks (Fig. 2a, b). The majority of BMDCs in the epidermis consisted of keratin-negative and langerin/CD207 (Langerhans cell marker)-positive cells (Supplementary Figure 7). However, a subset of keratin-expressing BMDCs was detected in tissues taken from no treatment, acetone-treated, and TPA-treated groups by double staining for GFP and pan-keratin or K14. K14 was expressed throughout the epidermis following TPA treatment (Fig. 2a, c, d). The greatest number of keratin-positive BMDCs was identified in TPA-treated tissues, and the number of keratin-positive BMDCs increased three-fold following long-term TPA treatment (Fig. 2a, c, d).

In addition, grouped and solitary keratin-positive BMDCs were detected primarily in the basal layer (germinal layer) but also in the suprabasal layer and HFs (Fig. 2c). There was no significant difference between untreated and acetone-treated groups (Fig. 2d). Taken together, these results demonstrate that TPA-mediated inflammation and/or hyperplasia resulted in recruitment of BMDCs to the epidermis, a subset of which were cytokeratin-positive. Cytokeratin-immunoreactive solitary BMDCs were detected primarily in the basal layer but could also be found throughout the epidermis. In chronic inflammation, a significantly greater number of both keratin-positive and negative BMDCs was recruited to chronic TPA-treated skin.

### BMCs migrate towards HMGB1 and KCs in invasion assays

To address potential mechanisms of BMC recruitment to the epidermis, we further studied the interaction of BMCs and epidermal KCs. We performed Matrigel invasion assays, and in preliminary experiments established cell number and incubation time for the assay. Controls included blank inserts, medium without serum, medium with serum but no cells as bait, and irrelevant cell type as bait (Swiss mouse 3T3). Test baits included three main potential recruiting factors: epidermal KCs, different concentrations of HMGB1, and stromal cell-derived factor 1alpha (SDF1a). HMGB1 is expressed in the epidermis of skin (Supplementary Figure 8a–c). HMGB1 expression in KCs is increased in UV-exposed skin and chronic inflammation-associated skin lesions, and is a known mediator of BMC-recruitment in skin injury^13,22,23^. In addition, SDF1 is associated with skin wound healing and is produced by dermal fibroblasts^24^.

As demonstrated in Supplementary Figure 8d, BMCs migrated through the Matrigel towards both epidermal KCs and HMGB1 significantly better than towards controls. Moreover, we observed an increase in HMGB1 immunoreactivity when epidermis was treated with TPA. Additional Matrigel invasion assays were performed to confirm the specificity of HMGB1 in BMC migration. The invasion assays were performed in the presence of HMGB1 with and without HMGB1 antibody for two different time points (24 and 30 h). The number of migrated BMCs was significantly decreased in the antibody-treated group, but not in the group treated with HMGB1 alone (Supplementary Figure 8e).

These findings suggest that HMGB1 may be one of the recruiting molecules produced by TPA-treated skin. These findings also suggest that epidermal cells or their secreted factors are themselves attractive to cultured BMCs. In contrast to the robust attraction of BMCs to HMGB1, SDF1a failed to elicit in vitro migration of BMCs. This observation was unexpected due to previous reports implicating SDF1a as a mobilizing factor for HSCs and MSCs^25^.

### BMDCs contributed to the epithelium of papillomas

**Fig. 3:**
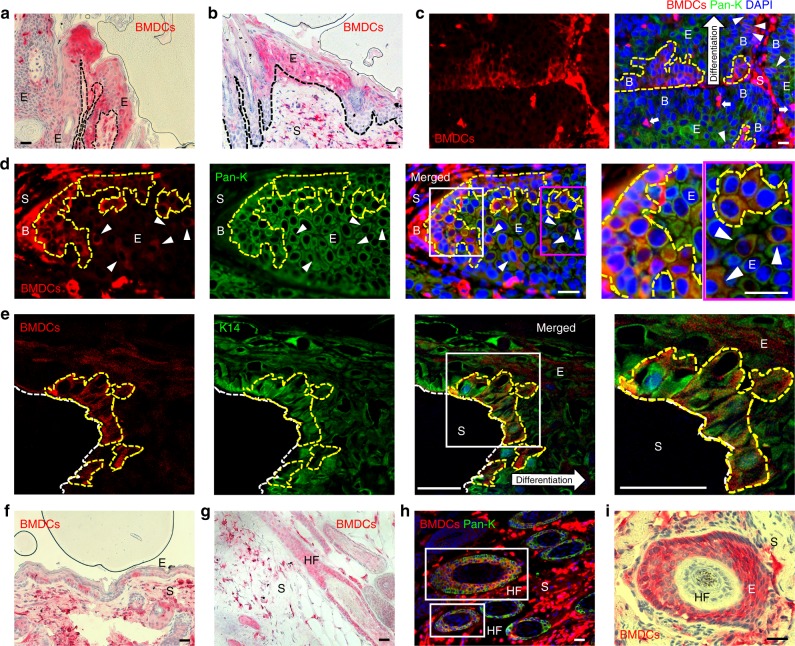
Keratin-expressing BMDCs are in the basal layer of the papilloma epithelium. **a**, **b** GFP-positive BMDCs (red) were detected in the **a** papilloma epithelium and **b** junctional area of papilloma and adjacent hyperplastic epidermis in paraffin sections of a papilloma. **c**, **d** Groups of pan-keratin-positive BMDCs (areas of yellow broken line and white arrowheads) were detected in basal parts of the papilloma epithelium in paraffin sections (keratin-negative BMDCs: white arrows). Areas in white and magenta box in figure d are magnified. **e** A group of K14-positive BMDCs (areas of yellow broken line) were detected in basal epithelium of frozen papilloma section (40 µm) using a multiphoton confocal microscope (note: tumor stroma was washed out in the staining processes). Area in white box is magnified. **f**, **g** BMDCs were detected in tumor-adjacent **f** hyperplastic epidermis, and **g** hair follicles. **h** Pan-keratin-positive BMDCs were identified in HFs (white boxes) under the tumor mass. **i** BMDCs were identified in deregulated HF structure under the tumor mass. *Black and white scale bars, 50 µm

To examine recruitment of BMDCs during skin tumor development, papilloma samples were collected from DMBA/TPA-treated BMT recipient mice (Supplementary Figure 5b). To detect the presence of BMDCs in the tumors, GFP-positive BMDCs were visualized directly from bisected unstained whole mounted tumors (Supplementary Figure 9). Immunohistochemical staining for GFP revealed BMDCs form single cells or small/large cluster groups in the epithelium of papillomas (Fig. 3a, b, i, Supplementary Figure 10). Moreover, groups of pan-keratin- and K14-immunoreactive BMDCs were detected in the basal (germinal) epithelium (Fig. 3c, d, e). To quantify the contribution of BMDCs, serial sections of 45 tumors from 21 mice in six different independent BMT groups were randomly chosen for immunohistochemical staining, and the area occupied by BMDCs in the epithelium of papillomas was measured. Solitary BMDCs were excluded from the quantification to avoid counting immune cells, which were usually detected as solitary GFP-positive cells within the epithelium. Groups of BMDCs were identified in 37.78% (17/45) of papilloma sections (Fig. 3a, b, i). Groups of BMDCs were confirmed by their location, morphology, and detection in multiple serial sections. In addition, 12 out of 45 papillomas were larger than 4 mm in diameter. BMDECs were identified in 25% (3/12) of large papillomas. In papillomas less than 4 mm in diameter, BMDCs were identified in 42.42% (14/33) of tumors. The total area occupied by BMDECs was measured in sections of GFP-positive tumors via AxioVision software (Zeiss). The average area of BMDC was 24.95% (*n* = 14, s.d. = ± 15.94) in the epithelium of BMDC-containing papillomas.

In addition, groups of BMDCs were also detected in hyperplastic epidermis adjacent to tumors (55.56%, 25/45 of tumor samples) (Fig. 3f–h). BMDCs were identified in both papillomas and adjacent skin from 30% (15/45) of papilloma samples. Although engraftment of BMDCs was not detected all tumor samples, these results demonstrate a significant contribution of BMDECs within the germinal layer of papillomas and suggest that these BMDECs are a critical source of cellular heterogeneity during tumor development.

### BMDCs contributed to ulcer-associated skin dysplasia

**Fig. 4:**
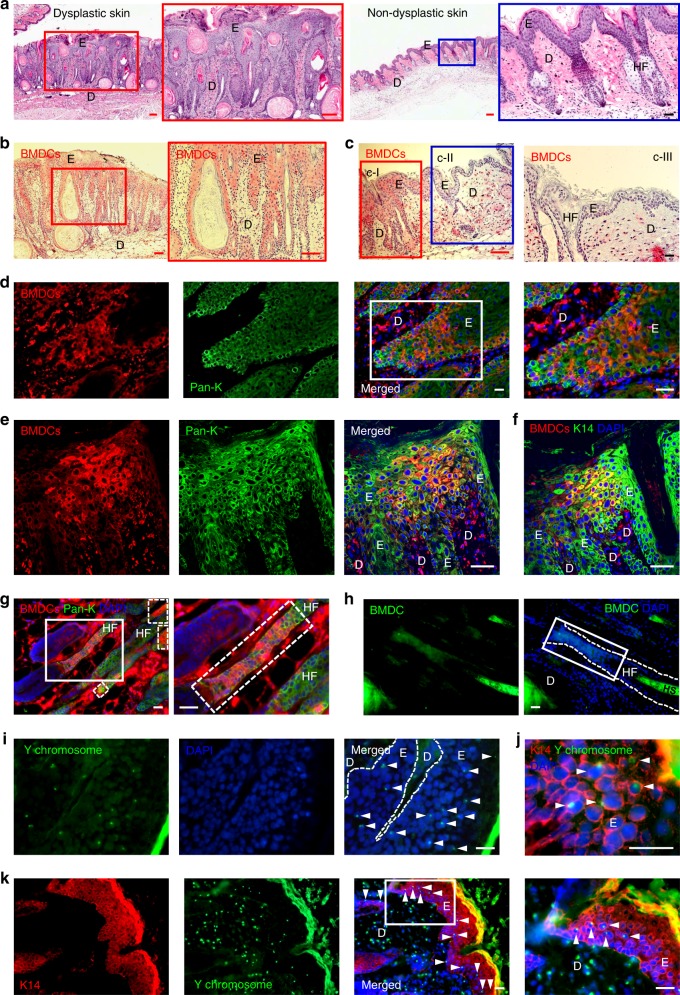
BMDECs are recruited in ulcer-associated dysplasia. **a** Dysplastic skin shows extremely abnormal epithelial structure (red box area is magnified). Non-dysplastic (mildly hyperplastic) epidermis adjacent to dysplasia (blue box area is magnified). **b** BMDCs (red) are observed in the dysplastic epithelium (red box area is magnified). **c** BMDCs (red) are identified in dysplastic epidermis (red box, c-I) but not in non-dysplastic-adjacent epidermis (blue box, c-II). BMDCs are not observed in non-dysplastic epidermis adjacent to dysplasia (c-III). **d** Clusters of keratin expressing BMDCs are identified in the basal region of dysplastic epithelium (white box area is magnified). **e**, **f** A significant contribution of **e** pan-keratin-expressing and **f** K14-expressing BMDECs are detected in dysplastic epithelium using a multiphoton confocal microscope. **g** Keratin-expressing BMDCs are identified in HF adjacent to a dysplastic wound (broken line boxes). White box area is magnified. **h** A group of GFP-positive cells (white box) are directly detected in HF in the dysplastic area of frozen skin sections using the GFP-channel. **i** The number of Y chromosome-positive donor-derived BMCs (white arrowheads) are identified in the frozen dysplastic skin section of female recipients. **j**, **k** K14/Y chromosome-positive (double positive) cells (white arrowheads) were identified in the dysplastic epithelium of frozen sections of female recipients. **j** A micrograph with high magnification and **k** a micrograph with low magnification of different samples and white box area is magnified. *Black and white scale bar, 50 µm; Red scale bar, 200 µm

Interestingly, unlike unirradiated controls, many DMBA-initiated C57BL/6 BMT recipient mice developed spontaneous skin ulcers upon long-term TPA promotion (Supplementary Figure 5c). These ulcers did not develop in FVB/N mice undergoing the same protocol. Histopathological samples of ulcers were reviewed by a pathologist, who found evidence of significantly increased epidermal hyperplasia compared with TPA-treated skin lacking ulcers. Evidence of dysplasia including cell elongation and abnormal epithelial structure was also noted in ulcer-containing tissue (Fig. 4a). To examine the contribution of BMDCs to the dysplastic epithelium, clusters of GFP-positive BMDCs were examined by staining for GFP, which revealed the presence of BMDCs in 53.06% (26/49) of dysplastic epidermis samples. Samples were collected from 14 mice in five different independent BMT groups (Fig. 4b, c). Approximately 35% (*n* = 22, s.d. = ± 18.86) of the entire dysplastic epithelium examined was determined to be BM-derived. Contribution of BMDCs was principally observed in ulcer-associated dysplastic epidermis but not in uninvolved epidermis (Fig. 4c, Supplementary Figure 10c). The area of BM-derived epithelium in ulcer-associated dysplasia (35%) was greater than in chemically-induced papillomas (24.95%). Most of the BMDCs in the epithelium were immunoreactive with pan-keratin and K14 antibodies and were observed in dysplastic areas and in hyperplastic epidermis adjacent to the ulcers (Fig. 4d–g, Supplementary Figure 10c, d). Additionally, we confirmed recruitment of BMDCs by direct visualization of GFP in frozen sections of dysplastic skin (Fig. 4h, Supplementary Figure 10e).

Fluorescence in situ hybridization (FISH) and immunostaining detected Y chromosome-positive and K14-positive epithelial cells in dysplastic tissues, confirming the recruitment of BMDCs within the epidermis (Fig. 4i–k). Moreover, we confirmed the Y-FISH with XY-FISH and found a notable absence of XXXY-reactive or XXXX-reactive nuclei, suggesting that if cell fusion occurs, it is not a major contributor to the phenomenon we report herein (Supplementary Figure 11a). Additional confirmation was performed using mathematical analysis after counting X and Y chromosomes from 2000 cells from XY-FISH samples. Mathematical analysis demonstrated that the probability of finding any tri/tetraploid cell in the samples is very close to zero (Supplementary Figure 11b). Furthermore, we investigated whether BMDECs in cutaneous tumors or dysplastic ulcers retained residual markers associated with BMCs (CD44 and CD45).

Despite using several different antibodies, immunoreactive cells were only detected from stromal cells, not in epithelial cells, including BMDECs (data not presented here). This could mean that transdifferentiation was completed, or that the BMDECs did not express either of those markers. These results demonstrated that a remarkable number of BMDECs engrafted into ulcer-associated dysplastic epithelium and were involved in the pathological progress of skin ulcer.

### BMDECs actively proliferated in chronic malignant wounds

**Fig. 5:**
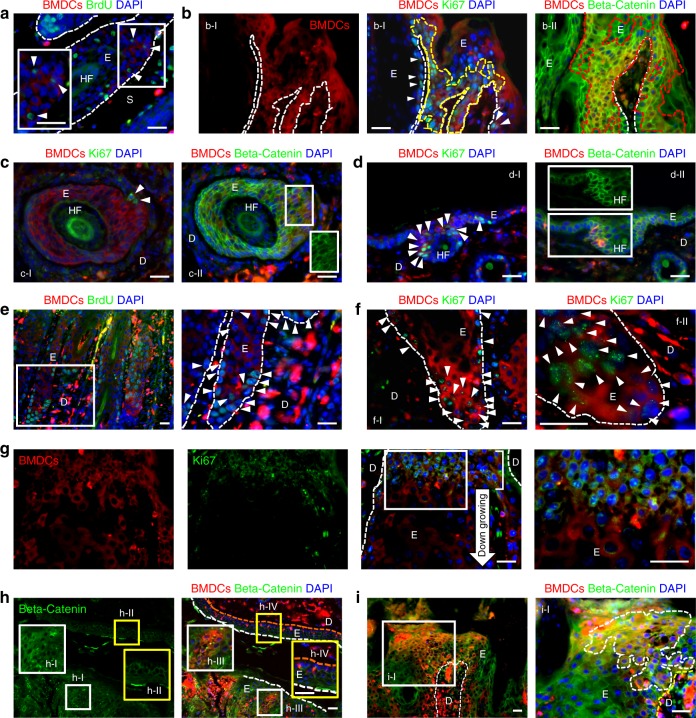
BMDECs in the papillomas and dysplasia are proliferating. **a** BrdU-positive BMDECs (arrowheads, white box area is magnified) are identified in the outer root sheath area of a deregulated HF under the tumor mass. **b** BMDECs in epithelium of papilloma (a serial section of Fig. 3a). b-I A group of Ki67-expressing BMDECs (area of yellow broken line and arrowheads) are identified in the basal epithelium of the papilloma. b-II Cytoplasmic beta-catenin expression was detected from BMDECs (areas of red broken line) in the epithelium of the papilloma. **c** BMDECs in deregulated HF structure under the tumor mass (a serial section of Fig. 3i). c-I Few Ki67-positive BMDCs (arrowheads) were identified in the section and c-II BMDCs in this area show membrane beta-catenin expression (GFP-channel in white box shows membrane expression). **d** BMDECs in tumor-adjacent hyperplastic epidermis (a serial section of Fig. 3f). d-I Ki67-expressing BMDCs (arrowheads) are located in HF and d-II strong membrane beta-catenin expression is detected overall in the epidermis, but low levels of cytoplasmic beta-catenin expression are detected in BMDCs in the HF (GFP-channel in white box). **e**–**i** BMDECs in dysplastic skin. **e** BrdU-positive BMDCs (arrowheads) are identified in the basal region of dysplastic epithelium. White box area is magnified. **f** f-I Ki67-expressing BMDCs (arrowheads) are identified in the basal region of dysplastic epithelium. f-II Higher magnification micrograph from different dysplastic skin sample. **g** Small Ki67-expressing BMDCs (white box area is magnified) are detected in the neck region (white line, located at junction between down-growing deregulated HF and interfollicular epidermis). **h** KCs and BMDCs in dysplastic epidermis (white boxes, magnified in h-I and h-III) show cytoplasmic beta-catenin expression, but cells in normal epidermis (yellow boxes, magnified in h-II and h-IV, orange broken line indicates basal layer of epidermis) show membrane expression. **i** BMDCs in dysplasia (white box area and area of white broken line in i-I) show cytoplasmic beta-catenin expression. *White scale bar, 50 µm

To demonstrate direct contribution of BMDECs at the recruited sites, the ability of BMDECs to proliferate in chronic wounds was examined by BrdU incorporation and Ki67 expression. Immunostaining with antibodies to BrdU and Ki67 revealed that BrdU- and Ki67-positive BMDECs were detected in the basal layer of papillomas (Fig. 5a, b, a serial section of Fig. 3a, Supplementary Figure 12a, b). Moreover, a large number of BrdU-immunoreactive and Ki67-immunoreactive BMDCs was found in the proliferative region of the dysplastic epithelium (Fig. 5e, f). Interestingly, unlike Ki67-positive cells in normal skin (Supplementary Figure 13), we identified a number of small Ki67-positive BMDECs, smaller than differentiated cells, in the neck region of epithelial down-growth structures (Fig. 5g). To characterize the active proliferation of BMDECs in the dysplastic epithelium, we examined beta-catenin expression in those BMDECs that showed membrane expression in non-proliferating KCs (Fig. 5h-II, h-IV) but cytoplasmic expression in proliferating cells (Fig. 5h-I, h-III). A high-level of cytoplasmic beta-catenin expression was detected among BMDECs in papillomas (Fig. 5b-II) and dysplastic epithelium (Fig. 5h-I, h-III, i). Interestingly, BMDECs showed a variable pattern of Ki67 and beta-catenin expression in different locations of the papillomas. The BMDECs in the epithelium of the tumor mass stained positively for both Ki67 and cytoplasmic beta-catenin (Fig. 5b, a serial section of Fig. 3a, Supplementary Figure. 12a, b). However, BMDECs in deregulated HF-like structures showed membrane expression of beta-catenin in areas with few Ki67-positive cells in the same tumor (Fig. 5c, a serial section of Fig. 3i, Supplementary Figure 12c, d). KCs and BMDECs in tumor-adjacent skin displayed primarily membrane beta-catenin expression; however, a few cells in the HF showed low cytoplasmic expression and Ki67 expression (Fig. 5d, a serial section of Fig. 3f, Supplementary Figure. 12e, f). In addition, we investigated whether BMDCs incurred the signature mutation (Ha-*ras* codon 61A to T transversion, CAA > CTA) characteristic of DMBA exposure. GFP-positive BMDCs were isolated from tumors and dorsal skin of BMT recipients, and sorted by FACS. Mutation detection was performed by nested PCR of DNA from GFP-positive cells followed by sequencing around codon 61 with the Ha-*ras* codon 61 mutation positive control (detailed method is described in the ref. ^1^). The signature mutation was not detected in any epithelial cells in chronic skin wounds (data not presented here). Taken together, these results strongly suggested that non-carcinogen-exposed BMDECs actively proliferated and contributed, at least in part, to the population of deregulated malignant epithelial cells in chronic skin wounds.

### Bulge stem cells contributed to ulcer-associated dysplasia

**Fig. 6:**
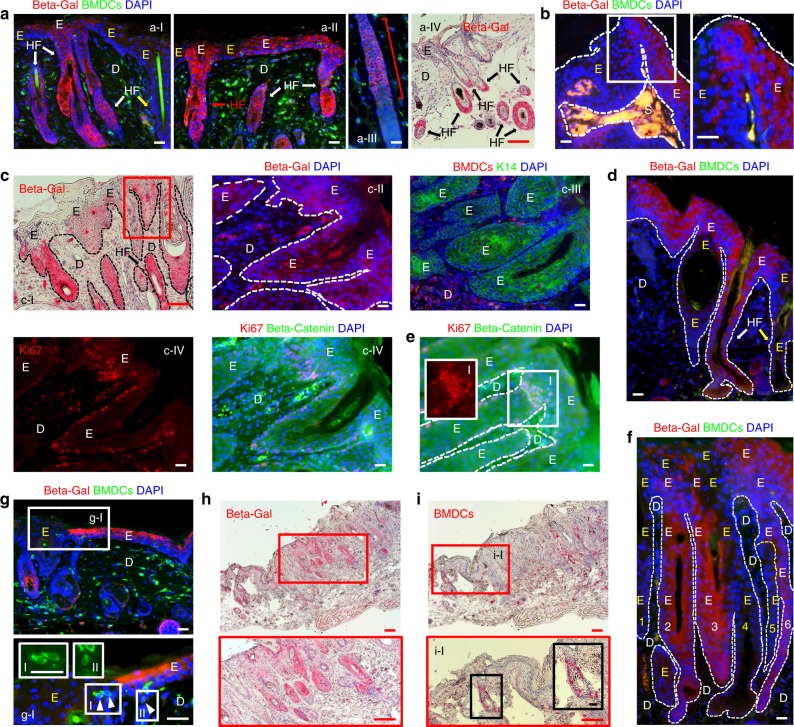
K15-positive bulge stem cells contribute to development of ulcer-associated dysplasia. **a** Contribution of K15-positive bulge stem cells (Beta-gal-positive) in hyperplastic epidermis. a-I Beta-gal-positive (white arrows and white E) and negative (yellow arrow and yellow E) HF are identified. a-II Beta-gal-positive cells are identified in HFs (red and white arrows) and interfollicular epidermis in hyperplastic epidermis. a-III Beta-gal-positive cells (red line) are identified in the bulge and lower HF in anagen. a-IV Beta-gal-positive cells (red IHC staining and black arrows) in HF bulge area and lower HF. **b** Contribution of Beta-gal-positive cells in papilloma epithelium. The junction of Beta-gal-positive and negative area (white box) is magnified. **c** Contribution of K15 bulge stem cells in dysplasia. c-II-IV Serial sections of red box area in c-I. c-I and c-II Beta-gal-positive cells are mainly located in the HF structure in dysplastic epidermis. c-III Serial sections show beta-gal-positive area is also K14-positive, and (c-IV) cytoplasmic beta-catenin expression with Ki67-positive cells located in basal region. **d** Beta-gal-positive cells located in both HF (white arrow) and interfollicular epidermis (white E) in dysplastic epidermis. **e** Ki67- and beta-catenin-expressing cells located in the neck region of dysplastic HF. **f** Cellular sources from six different dysplastic HFs (three beta-gal-positive, white E’s, and three beta-gal-negative, yellow E’s) participate in developing dysplastic interfollicular epidermis. **g** Beta-gal-positive cells (white E) and KC-shaped BMDCs (arrowheads) were observed together in hyperplastic interfollicular epidermis. White box area is magnified. g-I KC-shape BMDCs in white box-I and white box-II are magnified. **h**, **i** IHC of serial sections identified beta-gal-positive cells and BMDCs in close area of dysplastic epidermis. **h** Beta-gal-positive cells are in dysplastic HFs. Red box area is magnified. **i** A group of BMDCs were identified in a HF adjacent to dysplasia. Red box area is magnified. i-I Area of BMDCs is magnified further in black box. *Black and white scale bar, 50 µm; Red scale bar, 200 µm

To identify non-BM-derived cellular sources of ulcer-associated dysplasia, we hypothesized that K15-expressing bulge stem cells contribute to ulcer-associated dysplasia. To address this question, multistage skin carcinogenesis was performed in female *Krt1-15CrePR1;R26R* mice in which the progeny of K15-expressing cells in the HF bulge can be traced by detecting beta-galactosidase-expressing cells^26^. A subtumorigenic dose of DMBA treatment was followed by RU486 treatment of the dorsal skin of BMT recipients^1^. BMT was performed 1 week after RU486 treatment and TPA treatments followed 5 weeks after BMT (DMBA ► RU486 ► BMT ► TPA, Supplementary Figure 14). Confirming previous observations, immunostaining for beta-galactosidase revealed the contribution of beta-galactosidase-expressing bulge-derived cells in TPA-induced hyperplastic epidermis (Fig. 6a, Supplementary Figure 14a) and papillomas (Fig. 6b, Supplementary Figure 14b). These beta-galactosidase-positive cells were also identified in ulcer-associated dysplastic skin (Fig. 6c, d, f, h, Supplementary Figure 14b) and were observed primarily in the lower HF (Fig. 6a-III, a-IV) with the occasional entire HF in hyperplastic (Fig. 6a-I, a-II) and dysplastic epidermis being positive (Fig. 6d, h, f). Furthermore, the contribution of beta-galactosidase-positive cells to the interfollicular epidermis was often observed in TPA-induced hyperplastic skin (Fig. 6a-II, g) and dysplastic skin (Fig. 6d, f).

Similar to our observations that BMDCs in dysplastic skin stain positively for cytoplasmic beta-catenin, beta-galactosidase-, and K14-positive cells in dysplastic skin (Fig. 6c-I–III, serial sections of red box area in c-I) were also positive for cytoplasmic beta-catenin (Fig. 6c-IV, a serial section of red box area in c-I). A subset of these cells in the proliferating (basal and neck) region expressed Ki67 (Fig. 6c-IV, e). Moreover, BMDCs with KC morphology were identified near beta-galactosidase-positive cells in the hyperplastic interfollicular epidermis and HF in areas adjacent to dysplastic epithelium (Fig. 6g–i). Interestingly, our findings suggested that contribution of cells from multiple different HF origins contributed to the development of dysplastic interfollicular epidermis (Fig. 6f). Therefore, these results strongly suggested that stem cells involved in inflammation-mediated hyperplastic and dysplastic skin development were derived from a population of HF bulge stem cells as well as from BMDCs.

### Donor BMDCs initiated skin tumors from recipients after TPA

**Fig. 7:**
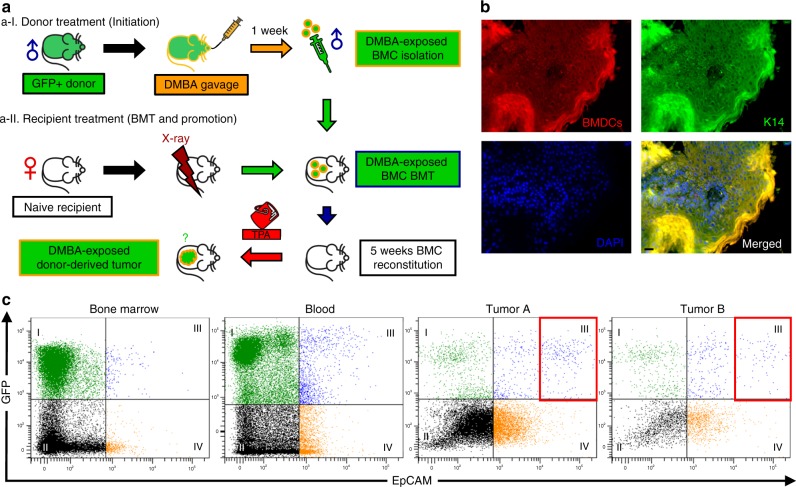
BMDCs initiate squamous lesions in the skin. **a** Overview of DMBA-exposed BMT to naive recipients: a-I Carcinogen-exposed donor BMC preparation: FVB/EGFP male donor mice were given one treatment of DMBA (1 mg in 0.5 ml corn oil) via oral gavage and DMBA-exposed whole toxic BMCs were isolated for BMT. a-II Naive female FVB/N mice were prepared as BMT recipients, and twice-weekly topical TPA (5 nmol for 3 weeks followed by 17 nmol in 200 µl of acetone until 18 weeks) promotion followed 5 weeks after BMT for 18 weeks (1. DMBA (BMT donor) ► 2. BMT ► TPA (BMT recipient)). Controls included corn oil/acetone, DMBA/acetone, and corn oil/TPA. None of the controls developed tumors. **b** K14-positive BMDCs identified from the squamous cell carcinoma sample from DMBA-exposed BMT recipient. All five of the tumors demonstrated co-expression of GFP and K14. Some heterogeneity was observed; however, most of the tumor epithelium was composed of cells of donor origin. Black scale bar, 50 µm. **c** Flow cytometric analysis: blood, bone marrow, and two disaggregated squamous tumor samples from DMBA-exposed BMT recipients. Native GFP fluorescence is shown in the *Y*-axis, and EpCAM, an epithelial cell marker, is shown along the *X*-axis. Note the GFP^+^/EpCAM^+^ double positive cells in quadrant III, and that there are more double positive cells in the blood than in the bone marrow. Note further, GFP^high^/EpCAM^high^ double positive cells are increased in both tumors (red boxes). The single EpCAM^+^ cells in quadrant IV suggest heterogeneity of the tumor epithelium. *Some images in Fig. 7a were generated with AutoDraw

To address the problem of BMC involvement in skin tumor initiation, we generated carcinogen-exposed BMCs by administering DMBA (1 mg in 0.5 ml corn oil) to donor mice (FVB/EGFP) by gavage, one week prior to BMT (Fig. 7a-I) from donors to naive female recipients (FVB/N, Fig. 7a-II). In a cohort of 15 females treated topically with TPA (5 nmol for first 3 weeks after BMT and 17 nmol later), four mice developed squamous neoplasms: three mice had one papilloma each, and one mouse had a papilloma and a squamous cell carcinoma. In addition, none of the control mice developed tumors. Immunofluorescence microscopy of each lesion disclosed that almost the entire epithelium was immunoreactive to GFP (Fig. 7b). Moreover, flow cytometry of the disaggregated tumors indicated that a subset was GFP-positive (native fluorescence), and a subset was GFP^+^/EpCAM^+^, but the GFP^high^/EpCAM^high^ population was only detected in tumor samples (Fig. 7c and Supplementary Figure 15). We noted with interest that the GFP^+^/EpCAM^+^ populations were present in both BM and blood, with numbers in blood slightly greater than those in BM. Possibly, this was due either to chronic mobilization of the cells from the BM to the blood, or perhaps to dissemination of lesional cells. Together, these results suggest that a subpopulation of BMDCs is recruited to the cutaneous epithelium upon tumor promotion and that they have the ability to initiate benign and malignant neoplasms.

## Discussion

We have reported here that in vivo, recruitment, epithelialization, and proliferation of BMDCs occurred during cutaneous carcinogenesis. In the multi-stage model of carcinogenesis, the skin is exposed to a single topical subthreshold dose of a carcinogen such as DMBA (initiation), followed by chronic, repetitive epidermal hyperplasia of sufficient magnitude, such as that induced by TPA (promotion)^27^. Initiation with DMBA causes permanent genetic changes including mutations in the Ha-*ras* gene^27^, whereas promotion causes chronic hyperplasia and elicits manifestation of neoplastic changes. The consequences of skin tumor initiation and promotion are benign squamous papillomas, some of which progress to malignancies such as squamous cell carcinomas, the principal lesions in mice. This model has been well characterized both biochemically and at the cellular level^28^, and is therefore useful for studying biological phenomena underlying mechanisms of carcinogenesis such as we report here.

The first observation that distinguishes our report from those previously published is the magnitude of BMDC recruitment: approaching 25% in many epithelial areas in over 40% of papillomas, and 35% of the lesional areas in 53% of the dysplastic ulcers studied. This contrasts with about 11% seen following acute wounding, and even less recruitment in normal cutaneous epithelium^10^. The magnitude of the recruitment observed here suggests a possible role in the process of skin carcinogenesis. Although DMBA initiation at the dosage used in this study was not sufficient to cause hyperplasia or inflammation of the skin, chronic long-term TPA treatment provided a significantly inductive environment for papillomas. In the case of the observed ulcers, the chronic wound-induced microenvironment might have elicited BMDC-mediated healing or survival responses supporting continuous proliferation. Alternatively, the high degree of inflammation could have been brought about by death of endogenous tissue stem cells, such as might have occurred upon x-irradiation and persistent challenge with TPA. We note that BMDC recruitment was observed in two published studies on *Helicobacter* infection of gastric mucosa, also apparently induced by damage leading to gastric ulcers followed by cancer^18,19^.

Our second observation differing from previous reports of acutely wounded tissue is the high level of BMDC epithelialization and keratinization. The mechanism underlying this observation is completely unknown at this time. Nevertheless, BMDECs were found not only in the germinative layers of papillomas and dysplastic ulcers, but also in the differentiated layers apparently contributing to the granular and cornified layers of the epithelium. Whether the BMDCs undergo a mesenchymal-to-epithelial transition, or whether they are derived from a rare BM-derived epithelial progenitor remains to be determined. Additionally, we cannot completely exclude the possibility of fusion or direct contact of BMDCs with endogenous epithelial cells. However, we submit that de novo epithelialization could have occurred as in our in vitro experiments in which plastic-adherent BMCs were separated from co-cultured KCs by an impassible filter. Moreover, additional evidence from XY-FISH result supports that the engrafted BMDECs are derived by direct transdifferentiation from BMC to KC.

Our third singular observation is the relatively high levels of proliferation of engrafted BMDECs (not exposed to carcinogen). As determined by BrdU incorporation, mKi67 expression, and cytoplasmic beta-catenin expression, it appeared that BMDECs in papillomas and ulcers had features of malignancy as seen in tumor cells. In this regard, we note with interest a paper from the Watt group^29^ demonstrating that non-dividing, differentiated epidermal cells could initiate tumor formation by way of infiltrating immune cells that reprogrammed beta-catenin and induced ectopic HFs. This finding suggested that a malignant microenvironment might induce deregulation of beta-catenin and promotion of malignant growth of BMDECs from a non-carcinogen-exposed donor. Consequently, the microenvironment may exacerbate the effect of a malignant initiating cell source and lead to dysplastic changes in the BMDECs. Moreover, the clusters of BMDECs observed in papillomas may have arisen from either multiple engrafted BMDCs or clonally by way of proliferation from single engrafted cells.

We have reported here that the in vitro induction of epidermal keratins by plastic-adherent BMCs occurred in the absence of direct cellular contact or fusion in KC co-cultures where soluble factors alone could pass through a filter. Soluble factors produced by KCs acted synergistically with BMP5 to enhance keratin-induction in BMDCs, suggesting the involvement of multiple environmental signals. These observations are substantiated by Matrigel invasion assays in which adherent BMCs migrated toward epidermal KCs as well as toward HMGB1, an alarmin produced and secreted by epidermal KCs in response to TPA treatment. Further investigation of the environmental cues as well as the inducible subpopulation(s) of BMDCs (such as PDGFR-alpha-positive BMCs^13^) is likely to be instructive.

Moreover, we confirmed the contribution of HF stem cells to the development of papillomas as well as ulcer-associated dysplasia by placing *Krt1-15CrePr;R26R* mice in protocols for BMT and chemical carcinogenesis. The existence of both progeny of *Krt1-15* bulge stem cells^1^ and BMDECs in the same papillomas as distinct subpopulations supports the notion that multiple cellular sources contribute to benign cutaneous neoplasms.

Our final significant finding is the demonstration that carcinogen-exposed toxic BMCs from a DMBA-treated donor are sufficient to initiate squamous cutaneous neoplasms when transplanted to naive recipients subsequently treated with the tumor promoter, TPA. This demonstrated the idea that carcinogen-exposed BMCs, when recruited to a favorable microenvironment, can initiate tumorigenesis. This experiment is also relevant to the seed and soil hypothesis^30^, where, in our experiment, the seeds were the BMDCs and the soil was the chronic inflammation-associated microenvironment in long-term TPA-treated skin. Furthermore, we also note with interest that the earliest papilloma observed (9 weeks) underwent malignant conversion, but that papillomas that arose at later intervals (12–17 weeks) remained as benign lesions. This is consistent with the idea that earlier arising papillomas having a greater risk of malignant conversion^31^.

We have reported here significant findings in the context of the classical murine model of chemically induced skin cancer: the recruitment, keratinization, stratification, and in situ proliferation of BMDCs in papillomas and dysplastic ulcers, as well as the keratinization of plastic-adherent BMCs in vitro in the absence of direct contact with epidermal KCs or fusion, and the contributions of BMDECs and the progeny of HF bulge stem cells to papillomas. The magnitude of the BMDC recruitment strongly suggests as a possible mechanism the damage or death of endogenous epithelial stem cells, as well as the likelihood of a gradient of soluble factors that is responsible for BMDC recruitment. Equally tantalizing is the possibility of an occult epithelial progenitor derived from the BM. Finally, it will be critical to determine how BMC recruitment is associated with the probability of malignant conversion of benign papillomas to carcinomas. In this regard, we would predict that they might be recruited to the high-risk papillomas described by Darwiche and associates^31^. However, at this time we cannot exclude the possibility that they might play a protective role in papillomas of low risk of conversion. We are currently exploring solutions to these problems.

Furthermore, we identified tumors both with and without BMDECs. This indicates that the different microenvironments of these two groups of tumors may be influenced by the presence of BMDECs. Previous studies have demonstrated that BM-derived MSCs are associated with tumor progression and mediation of immune checkpoints^32–34^. Therefore, identifying BMDEC-favorable and non-favorable tumor microenvironments might provide diagnostic and prognostic markers as well as therapeutic approaches.

In addition, higher levels of the EpCAM^+^ population were detected in the blood, rather than the BM, of tumor-bearing toxic BMT recipients. This population may come from the BM and/or the tumor (GFP^+^/EpCAM^+^), or from GFP^−^/EpCAM^+^ populations from various organs including the GFP^−^/EpCAM^+^ population in the tumor. This result demonstrates the systemic contribution of an EpCAM^+^ population from the tumor as well as from multiple organs. Thus, this may indicate a higher level of trafficking between organs and the heterogeneous population of epithelial cells in the presence of chronic inflammation, which may cause noise, making it harder to identify circulating tumor cells using epithelial cell markers (e.g., EpCAM or cytokeratins)^35^.

We conclude that large numbers of BMDECs are recruited to a subset of cutaneous papillomas and that dysplastic ulcers reflect a previously unrecognized systemic contribution to these epithelial lesions. The implications of our findings toward understanding the etiology of epithelial cancers as well as their diagnosis and treatment have not escaped our notice. Although such studies are currently in progress, our documentation of BM-derived, proliferating, stratifying, and keratinizing cells in lesional cutaneous epithelium represents a significant advance in the fields of epithelial biology and cancer research.

## Methods

### Animals

Wild type C57BL/6 mice (male and female, 7–9 weeks old) were purchased from Taconic (Hudson, NY) and Charles River (Wilmington, MA). FVB/N mice (male and female, 7 weeks old) were purchased from Charles River (Wilmington, MA). Female *Krt1-15CrePR1;R26R* mice reported previously^26^ were bred at Columbia University. Chicken β-actin enhanced green fluorescence protein (EGFP) transgenic mice (male, 8 weeks old, C57BL/6 and FVB/N), human ubiquitin C green fluorescent protein (GFP) transgenic mice (male, 8 weeks old,) were purchased from Jackson Laboratory (Bar Harbor, ME). Animals were maintained in the AAALAC accredited Columbia University Barrier Facility, The Hormel Institute Animal Facility, and in the NIEHS animal facility under protocols approved by the Institutional Animal Care and Use Committee. All three animal facilities were AAALAC accredited. All animals were maintained in accordance with institutional and government guidelines for the care and use of experimental animals.

### Chemicals

7,12-Dimethylbenz[*a*]anthracene (DMBA), and 12-*O*-tetradecanoylphorbol-13-acetate (TPA) were purchased from Sigma (St. Louis, MO). Acetone was spectral grade from Fisher Scientific (Norcross, GA). DMBA and TPA were dissolved in acetone for cutaneous multi-stage carcinogenesis. DMBA was dissolved in corn oil for oral gavage. RU486 was purchased from Sigma as a powder and mixed at a concentration of 1 mg/g with Neutrogena Hand Cream (unscented) for topical application. Bromodeoxyuridine (BrdU, Sigma-Aldrich) was prepared in saline to deliver 50 µg/dose.

### BMC isolation

Whole BMCs were harvested from femurs and tibiae of 7- to 9-week-old male mice (C57BL/6, Chicken beta-actin EGFP, UBC-GFP, or FVB.Cg-Tg(CAG-EGFP)B5Nagy/J). The BMCs were isolated by flushing the femurs and tibiae with phosphate buffered saline (PBS) containing 1% fetal bovine serum (FBS) under sterile conditions using 25G needles. Isolated BMCs were resuspended in PBS solution for BMT, or mouse MSC culture medium (MesenCult medium, Stem Cell Technologies, Vancouver, BC, Canada) for subsequent culture. In order to culture adherent BMCs, red blood cells were removed using ACK lysing buffer (Lonza, United States).

### Bone marrow transplantation

C57BL/6 and FVB/N female mice underwent allogenic gender-mismatched BMT with genetically marked (GFP or EGFP) whole BMCs from male donors.

i) In order to demonstrate BMC recruitment (Supplementary Figure 5, 14), 9-week-old C57BL/6 female recipient mice (DMBA-pre-treated) were lethally irradiated (900 rads) twice with a cesium irradiation device (Mark I Irradiator, using Cs-137; JL Shepherd, San Fernando, CA). Whole BMCs were isolated from 8-week-old male genetically labeled transgenic mice (e.g., Chicken β-actin EGFP or UBC-GFP mice). Three hours after the second irradiation, 5 × 10^6^ GFP-labeled BMCs were infused via tail vein injection in each recipient. For bone marrow reconstitution, the recipient mice were allowed to recover for 5 weeks with food and antibiotic water (100 mg/l neomycin and 10 mg/l polymixin B sulfate).

ii) In order to demonstrate cancer initiation from BMCs (Fig. 7a), donor mice (FVB/EGFP) were pre-exposed to DMBA via oral gavage and whole BMCs were isolated 1 week after DMBA treatment. Isolated BMCs were transplanted to 8-week-old naive female recipients (FVB/N).

### Skin carcinogenesis

To demonstrate BMC recruitment (Supplementary Figure 5, 14), the dorsal fur of BMT recipient mice was clipped with electric clippers. One week after removing the dorsal fur, the mice received a single sub-tumorigenic dose of DMBA (200 nmol in 200 µl of acetone) applied to their dorsal skin prior to BMT. Five weeks after BMT, the mice received thrice-weekly treatments with the tumor promoter TPA (17 nmol in 200 µl of acetone). Skin or papilloma samples were collected from euthanized mice at different intervals (4 and 9 weeks, or 0, 5, and 15 weeks or more) during promotion. Treatment groups and controls are described in Supplementary Figure 5, 6, and 14.

To demonstrate cancer initiation from BMCs (Fig. 7a), male donor mice (FVB/EGFP) were administered DMBA (1 mg in 0.5 ml corn oil) via gavage. One week later, their DMBA-exposed whole toxic BMCs were transplanted to 8-week-old naive female recipients (FVB/N). After reconstitution for 5 weeks, recipient mice were treated topically twice weekly with TPA (5 nmol for 3 weeks followed by 17 nmol in 200 µl of acetone) for 18 weeks.

According to the conventional protocol BM reconstitution of BMT recipient is complete by 3 weeks, and usually treatment begins 3 weeks after BMT. However, skin carcinogenesis requires long-term TPA treatment. Therefore, to increase survival rate of the BMT recipient mice during TPA promotion, an extra 2 weeks were given for full recovery. The health of the mice was monitored three times per week by laboratory staff, and daily by veterinary staff.

### Keratinocyte harvest

KCs were harvested from dorsal skin of female C57BL/6 mice (7–9 weeks old) for BMC/KC co-culture. To detect GFP-positive BMDCs, KC were harvested from dorsal skin and papillomas from DMBA/TPA- or TPA-treated BMT recipient mice. To isolate KCs, euthanized mice were clipped of dorsal fur and decontaminated by washing with povidone iodine solution followed by 70% ethanol. The dorsal skin was dissected and the subcutaneous fat and muscle removed by scraping with a scalpel. One by 1.5 cm pieces of skin were floated hairy side up onto 0.25% trypsin for 2 h at 32 °C. The skin and hairs were then scraped from the dermis with a scalpel into SMEM with 10% FBS and then stirred at 100 RPM for 20 min at room temperature. The epidermis and hairs were filtered out with a 70 µm filter, filtrate was centrifuged and resuspended in SMEM with 10% FBS, and the resultant cells were counted with a hematocytometer for FACS or in vitro applications. All detail procedures have been described in our previous study^36^.

### BMC/KC co-culture

To induce BMC differentiation into KC, BMC/KC co-culture was performed. KCs were harvested from 7-week-old to 9-week-old C57BL/6 female mouse dorsal skin and cultured in KC medium (SPRD-111^37^) on the membranes of inserts (Transwell, pore size 0.4 μm; Corning, Lowell, MA) for five days until ~60% confluent, then switched to MesenCult medium. BMCs were harvested from 7–9-week-old C57BL/6 male mice by flushing femurs and tibiae with 1% FBS DPBS or Hank’s balanced salt solution (HBSS), and seeded on 6-well plates (Corning, Lowell, MA) in MesenCult medium. Non-adherent cells were removed 48 h after cell seeding, and BMC culture medium was changed every three days. One week after KC harvest, BMC culture (6-well plate) and KC culture (insert) were combined in the presence of Mesencult medium. Before combining, to remove residual KC medium, the inside and bottom of the insert were washed with PBS. For BMP5 (R&D systems, Minneapolis, MN; Cat# 6176-BM/CF) treatment, mesenchymal stem cell medium was changed every three days for 10 days, and BMP5 (50 ng/ml) was added immediately after changing medium (Supplementary Figure 1).

### Chemotaxis cell migration assay

Migration assays were performed in a migration chamber (Millipore, Burlington, MA) according to the manufacturer’s directions using epidermal KCs, Swiss mouse 3T3 cells (ATCC, Manassas, VA), HMGB1 (BioLegend, San Diego, CA; #764004), or SDF1a (R&D Systems, Minneapolis, MN; Cat# 460-SD/CF) as bait. Positive controls were epidermal KCs, and negative controls were included medium without serum or growth factors. In addition, to confirm the specificity of HMGB1 as bait, HMGB1 antibody (Invitrogen, Carlsbad, CA; #PA5-29604) was added in the presence of HMGB1 (10 and 50 ng/ml), and non-antibody-treated HMGB1 group served as a positive control. Preliminary experiments were performed to establish optimum dosage and migration times, and experiments were conducted with triplicate wells and were repeated three times.

### Tissue preparation

Skin tumor and tumor-adjacent uninvolved skin samples were collected after euthanizing the DMBA/TPA- or TPA-treated BMT recipient mice by CO_2_ asphyxiation followed by cervical dislocation and either fixed overnight in 10% neutral buffered formalin to make paraffin sections, or snap frozen in liquid nitrogen and embedded in Tissue-Tek OCT compound (Sakura Finetek, Torrance, CA) to prepare frozen sections. In addition, to detect active cycling cells in tissues, BrdU solution (50 µg/g body weight) was injected intraperitoneally to mice 1 h prior to euthanizing and the presence of BrdU was detected by anti-BrdU antibody.

### Flow cytometry

Cells were harvested from papillomas and a carcinoma from carcinogen-exposed toxic BMT recipients. Flow cytometry was conducted on a FACSAria II (BD Bioscience, San Jose, CA) using native GFP fluorescence and directly conjugated EpCAM antibody.

### Immunostaining

Paraffin and OCT-embedded tissues samples were sectioned to 5–8 µm thickness and mounted on positively charged microscope slides. Paraffin sections were deparaffinized with xylene and rehydrated with graded alcohols and water. Antigens were unmasked with citrate-based antigen retrieval solution (Vector Labs, Burlingame, CA). Frozen sections were fixed with chilled methanol and acetone. For immunofluorescence staining, sections were pre-incubated for 1 h at room temperature with 10% normal goat or horse serum (Vector Laboratories, Burlingame, CA) to block non-specific antibody binding. In addition, fish serum-based blocking reagent (Aves, Tigard, Oregon) was purchased for use with chicken primary antibody. After blocking, tissue sections were incubated with primary antibody in PBS (1% normal goat or horse serum) at 4 °C overnight. On the following day, tissue sections were incubated with secondary antibodies in PBS (1% normal goat or horse serum) for 1 h at room temperature. After washing, tissue sections were mounted with DAPI containing mounting medium (Vector labs, Burlingame, CA) and cover-slipped prior to fluorescence microscopy. Immunohistochemistry was performed with Vectastain ABC Kit (Vector Laboratories, Burlingame, CA) following the manufacturer’s provided protocol. Vector M.O.M. (Mouse on Mouse) immunodetection kits (Vector Laboratories, Burlingame, CA) were used following the manufacturer’s directions for mouse primary monoclonal antibodies on mouse tissues.

To detect keratin expression from co-cultured BMCs, cultured BMCs were washed with cold PBS and fixed with chilled methanol and acetone. The subsequent steps were performed as for the immunofluorescence staining process previously described. After staining, the bottom part of the BMC co-cultured cell culture dish was cut out using a rotary tool (Dremel, WI, United States) after immunostaining. The bottom of the cell culture dish was placed cell-side-down onto a large format cover glass (Thermo Scientific, Portsmouth, NH, Gold Seal cover glass 48 × 60 mm) and mounted with a plastic lantern-slide mount (Gepe, Zug, Switzerland, Size 6 × 4.5 cm) for subsequent light microscopy.

### X and Y chromosome detection

Snap frozen skin or tumor tissues were sectioned to 5 µm thickness. Sections were fixed with chilled ethanol (95%) for 10 min and dried at room temperature for 10 min. Fixed sections were denatured with 70% formamide (Sigma) at 73 °C for 5 min and dehydrated with graded alcohols. Prepared mouse Y chromosome probe (Cambio, StarFISH, Cambridge, UK) was applied on the dried sections and cover-slipped. Cover-slipped sections were sealed with rubber cement and preheated at 82 °C for 2 min. Preheated samples were placed in the humidified hybridization chamber at 45 °C for 14–20 h. After hybridization, cover slips were removed gently, and the sections were washed twice with 0.4× SSC containing 0.3% NP-40 (Abbott Molecular, Visys, Abbott Park, IL) at 73 °C for 2 minutes and with 2× SCC containing 0.1% NP-40 at 73 °C for 2 min. After washing, tissue sections were completely dried in the dark and mounted with DAPI-containing mounting medium (Vector Labs, Burlingame, CA). Cover-slipped sections were sealed with clear nail polish and stored at −20 °C in the dark. The control slides included tumors from male and female mice, including sections from mice previously demonstrated to express the Y chromosome in tumor epithelium. The test group consisted of sections from BMT mice. XY-FISH (Invitrogen, Carlsbad, CA) was performed according to the manufacturer’s directions.

### Analysis of the true probability of observing a tri/tetraploid cell

Notation

*n* is the number of independent experiments (Number of cells viewed).

*k* is the number of successes (Number of tri/tetraploid cells observed in the set of *n* cells studied).

*p* is the probability of success (observing a tri/tetraploid cell) in a single experiment.

While we do not know the value *p*, we can obtain a good estimate by answering the question: given the probability, *p*, of observing either a triploid or tetraploid cell, what is the probability of observing at least one triploid or tetraploid cell in a set of *n* independent cells? We answer this question using Equations (1–3) below.

The binomial distribution probability function (also called the probability density function) is given by:

1 $$\Pr \left( {k,n;p} \right) = \left( {\begin{array}{*{20}{c}} n \\ k \end{array}} \right)p^k(1 - p)^{n - k}$$

where $$\left( {\begin{array}{*{20}{c}} n \\ k \end{array}} \right) = \frac{{n!}}{{\left( {n - k} \right)!k!}}$$ is the binomial coefficient. Also, Pr(at least *k*_0_ successes) = Pr(Observing at least *k*_0_ tri/tetra-ploid cells when viewing *n* cells), where 0 ≤ *k*_0_ ≤ *n*, is given by:

2 $$\mathop {\sum }\limits_{k = k_0}^n \Pr \left( {k,n;p} \right) = \mathop {\sum }\limits_{k = k_0}^n \left( {\begin{array}{*{20}{c}} n \\ k \end{array}} \right)p^k(1 - p)^{n - k}$$

It follows that Pr(observing at least one tri/tetraploid cell when viewing *n* cells) is given by:

3 $$\mathop {\sum }\limits_{k = 1}^n \left( {\begin{array}{*{20}{c}} n \\ k \end{array}} \right)p^k(1 - p)^{n - k} = 1 - \left( {\begin{array}{*{20}{c}} n \\ 0 \end{array}} \right)p^0\left( {1 - p} \right)^n = 1 - \left( {1 - p} \right)^n$$

The 99% confidence interval of the true probability of the probability *p* was computed using this method implemented in the BINOM program^38^.

### Antibodies

Primary antibodies included: Chicken anti-GFP (1:1000; Aves, Tigard, Oregon; #GFP-1020), Rabbit anti-GFP (1:300; Invitrogen, Carlsbad, CA; #A11122) Rabbit anti-Pan-keratin (ready to use, Dakocytomation, Carpinteria, CA; #Z0622), Mouse anti-Mouse K15 (1:200; Thermo scientific, Fremont, CA; #MS1068-P), FITC-Rat anti-mouse CD34 (1:200; BD Pharmingen, San Diego, CA; #553733), Rabbit anti-Mouse K14 (1:1000; Covance, Princeton, NJ; #PRB-115P), Rat anti-Mouse CD207/Langerin (1:200; Dendritics, Lyon, France; #AB-IN786758), Rat anti-BrdU (1:100; Accurate chemical & Scientific, Westbury, NY; #OBT0030), Rat anti-Mouse CD44 (1:30; BD Pharmingen, San Diego, CA; #553132), Rabbit anti-Mouse Ki67 (1:200; Abcam, Cambridge, MA; #AB15580), Mouse anti-Mouse Beta-catenin (1:700; BD Pharmingen, San Diego, CA; #BD610153), Rabbit anti-BMP5 (Discontinued, Santa Cruz Biotechnology, Dallas, TX), Rabbit anti-Beta-Galactosidase (1:2000; Abcam, Cambridge, MA; #AB616).

Secondary antibodies were purchased from Life Technologies at dilution of 1:1000, and included: Alexa Fluor 488 Goat anti-Mouse IgG (#A11029), Alexa Fluor 488 Goat anti-Rabbit IgG (# 911008), Alexa Fluor 488 Goat anti-Rat IgG (#911006), Alexa Fluor 488 Goat anti-Chicken IgG (#911039), Alexa Fluor 594 Goat anti-Mouse IgG (#911005), Alexa Fluor 594 Goat anti-Rabbit IgG (#911012), Alexa Fluor 594 Goat anti-Rat IgG (#011007), Alexa Fluor 594 Goat anti-Chicken IgG (#911042).

### RNA extraction and quantitative RT-PCR analysis

Total RNA was extracted from co-cultured BMCs and control cells using TRIzol (Invitrogen, Carlsbad, CA). High-fidelity cDNA was produced form extracted RNA samples with the SuperScript III First-Strand Synthesis System (Invitrogen, Carlsbad, CA). Quantitative real time-PCR sample mixtures were prepared with QuantiTect SYBR Green PCR kit (Qiagen, Germantown, MD) based on the provided manufacturer’s protocol. Real time-PCR reactions were performed using an Applied Biosystems Prism 9700 PCR machine. The real-time PCR conditions were as follows: 95 °C for 15 min followed by 45 cycles of (95 °C for 15 s, 55 °C for 30 s, and 72 °C for 30 s).

Results from Q-RT-PCR were analyzed using relative quantitation method. Ct values of different genes were normalized to the house keeping gene (GAPDH) by subtracting Ct values, and ∆Ct values were calculated. The mean ∆Ct values were converted into fold change gene expression. Final values were normalized with log_2_ transformation. *P* value was determined by Fisher’s ANOVA method. Primer sequences were as follows: K14 Forward: 5-AGAGGACGCCCACCTTTCATCTTC-3 and Reverse: 5-CTCTTCCAGCAGTATCTGCGTCCAC-3; GAPDH Forward: 5-AAATGGTGAAGGTCGGTGTGAACG-3 and Reverse: 5-TCACACCCATCACAAACATGGGG-3.

### Microscopy

Prepared slides were viewed with a Zeiss Axioplan or Axio Imager microscope (Carl Zeiss, Thornwood, NY) equipped with Plan Neofluar objectives, and fluorescence, phase, and bright field optics. Photographs were taken with the accompanying Zeiss AxioCam digital camera. Image analysis was performed with Zeiss AxioVision software. Prepared slides were viewed with a Zeiss LSM 510 NLO multiphoton microscope (Carl Zeiss, Thornwood, NY) equipped with various objectives.

### Quantitative microscopy

Keratin-positive BMCs were counted from the entire surface of the culture dishes, counting cells that were both keratin- (cytoplasm) and DAPI- (nucleus) positive. Keratin-positive cells were detected with a 5× objective, and all keratin-positive cells were verified with 10× and 20× or higher magnification objectives.

BMDC and BMDECs were identified from tissue samples. Twenty different areas were randomly selected from each sample slide using a 20× objective. All counted GFP- (single) and GFP/Keratin- (double) positive BMDCs were DAPI-positive and located in the epidermis. All counted BMDCs were confirmed with 40× or higher magnification objectives.

To measure bone marrow-derived epithelial area, IHC staining with GFP antibody was performed with medial sagittal sections of papillomas and ulcer-associated dysplasia as determined by the section from 10 or more serial sections showing broadest attachment of the tumor or ulcer to the underlying stroma. The GFP-positive area consisted of at least five adjacent GFP-positive cells in the cutaneous epithelium. GFP-positive epithelial areas in the samples were confirmed via independent IHC staining of serial sections. Micrographs of samples were taken with 10× objective and were tiled manually. The percentage of GFP-positive area was carefully measured and computed with Zeiss AxioVision software.

### Statistical analysis and reproducibility

All in vitro and in vivo data were subjected to statistical analysis performed and confirmed by bio-statisticians. Significance of different groups of treatment and treatment time was determined by Student’s *t*-test, Fisher’s ANOVA and *F*-statistic. At least three different investigators tested samples and at least one person was blinded as to the assessment of the slides. The investigators were not blinded for animal treatment. All in vitro and in vivo assays were replicated by different people from two different institutions.

## Electronic supplementary material

Supplementary Information

## Data Availability

All data are available from the corresponding author upon request.

## References

[1] Li, S. et al. A keratin 15 containing stem cell population from the hair follicle contributes to squamous papilloma development in the mouse. *Mol. Carcinog*. **52**, 751–759 (2013). 10.1002/mc.21896 PMC3424369 22431489

[2] White AC (2011). Defining the origins of Ras/p53-mediated squamous cell carcinoma. Proc. Natl Acad. Sci. USA.

[3] Lapouge G (2011). Identifying the cellular origin of squamous skin tumors. Proc. Natl Acad. Sci. USA.

[4] Jang YY, Collector MI, Baylin SB, Diehl AM, Sharkis SJ (2004). Hematopoietic stem cells convert into liver cells within days without fusion. Nat. Cell Biol..

[5] Sasaki M (2008). Mesenchymal stem cells are recruited into wounded skin and contribute to wound repair by transdifferentiation into multiple skin cell type. J. Immunol..

[6] Krause DS (2001). Multi-organ, multi-lineage engraftment by a single bone marrow-derived stem cell. Cell.

[7] Poulsom R (2003). Bone marrow stem cells contribute to healing of the kidney. J. Am. Soc. Nephrol..

[8] Jiang Y (2002). Pluripotency of mesenchymal stem cells derived from adult marrow. Nature.

[9] Badiavas EV, Abedi M, Butmarc J, Falanga V, Quesenberry P (2003). Participation of bone marrow derived cells in cutaneous wound healing. J. Cell. Physiol..

[10] Brittan M (2005). Bone marrow cells engraft within the epidermis and proliferate in vivo with no evidence of cell fusion. J. Pathol..

[11] Borue X (2004). Bone marrow-derived cells contribute to epithelial engraftment during wound healing. Am. J. Pathol..

[12] Kataoka K (2003). Participation of adult mouse bone marrow cells in reconstitution of skin. Am. J. Pathol..

[13] Tamai K (2011). PDGFRalpha-positive cells in bone marrow are mobilized by high mobility group box 1 (HMGB1) to regenerate injured epithelia. Proc. Natl Acad. Sci. USA.

[14] Janin A (2009). Donor-derived oral squamous cell carcinoma after allogeneic bone marrow transplantation. Blood.

[15] Avital I (2007). Donor-derived human bone marrow cells contribute to solid organ cancers developing after bone marrow transplantation. Stem Cells.

[16] Worthley DL (2009). Human gastrointestinal neoplasia-associated myofibroblasts can develop from bone marrow-derived cells following allogeneic stem cell transplantation. Stem Cells.

[17] Cogle CR (2007). Bone marrow contributes to epithelial cancers in mice and humans as developmental mimicry. Stem Cells.

[18] Houghton J (2004). Gastric cancer originating from bone marrow-derived cells. Science.

[19] Varon C (2012). Helicobacter pylori infection recruits bone marrow-derived cells that participate in gastric preneoplasia in mice. Gastroenterology.

[20] Leder A, Kuo A, Cardiff RD, Sinn E, Leder P (1990). v-Ha-ras transgene abrogates the initiation step in mouse skin tumorigenesis: effects of phorbol esters and retinoic acid. Proc. Natl Acad. Sci. USA.

[21] Kangsamaksin T, Morris RJ (2011). Bone morphogenetic protein 5 regulates the number of keratinocyte stem cells from the skin of mice. J. Invest. Dermatol..

[22] Johnson KE, Wulff BC, Oberyszyn TM, Wilgus TA (2013). Ultraviolet light exposure stimulates HMGB1 release by keratinocytes. Arch. Dermatol. Res..

[23] Hoste E (2015). Innate sensing of microbial products promotes wound-induced skin cancer. Nat. Commun..

[24] Bollag WB, Hill WD (2013). CXCR4 in epidermal keratinocytes: crosstalk within the skin. J. Invest. Dermatol..

[25] Iinuma S (2015). Transplanted bone marrow-derived circulating PDGFRalpha + cells restore type VII collagen in recessive dystrophic epidermolysis bullosa mouse skin graft. J. Immunol..

[26] Morris RJ (2004). Capturing and profiling adult hair follicle stem cells. Nat. Biotechnol..

[27] Abel EL, Angel JM, Kiguchi K, DiGiovanni J (2009). Multi-stage chemical carcinogenesis in mouse skin: fundamentals and applications. Nat. Protoc..

[28] Yuspa SH (2000). Overview of carcinogenesis: past, present and future. Carcinogenesis.

[29] Baker CM, Verstuyf A, Jensen KB, Watt FM (2010). Differential sensitivity of epidermal cell subpopulations to beta-catenin-induced ectopic hair follicle formation. Dev. Biol..

[30] Paget S (1889). The distribution of secondary growths in cancer of the breast. Lancet.

[31] Darwiche N (2007). Expression profile of skin papillomas with high cancer risk displays a unique genetic signature that clusters with squamous cell carcinomas and predicts risk for malignant conversion. Oncogene.

[32] Hall B., Andreeff M. & Marini F. The participation of mesenchymal stem cells in tumor stroma formation and their application as targeted-gene delivery vehicles. *Handbook Exp. Pharmacol.* **180**, 263–283 (2007). 10.1007/978-3-540-68976-8_12 17554513

[33] Karnoub AE (2007). Mesenchymal stem cells within tumour stroma promote breast cancer metastasis. Nature.

[34] Martinet L (2009). A regulatory cross-talk between Vgamma9Vdelta2 T lymphocytes and mesenchymal stem cells. Eur. J. Immunol..

[35] Morris RJ (2017). Circulating tumor cells: quintessential precision oncology presenting challenges for biology. npj Precis. Oncol..

[36] Wu WY, Morris RJ (2005). Method for the harvest and assay of in vitro clonogenic keratinocytes stem cells from mice. Methods Mol. Biol..

[37] Morris, R. J. & Fischer, S. M. in: *Keratinocyte Methods and Keratinocyte Handbook: The Keratinocyte Handbook* (eds Leigh, I. M. et al.) (Cambridge University Press, Cambridge, 1994).

[38] Ott, J. *Analysis of Human Genetic Linkage*, 3rd edn. (Johns Hopkins University Press, 1999).

