# Supplementary material for: Bone marrow-derived epithelial cells and hair follicle stem cells contribute to development of chronic cutaneous neoplasms
Source: Nat Commun. 2018 Dec 13;9:5293. doi: 10.1038/s41467-018-07688-8 (PMC6294255; doi:10.1038/s41467-018-07688-8)
Supplement: Supplementary file 1 — Supplementary Information [file 41467_2018_7688_MOESM1_ESM.pdf]

**Supplementary Information for:**

# Bone marrow-derived epithelial cells and hair follicle stem cells contribute to development of chronic cutaneous neoplasms

Park *et al.*

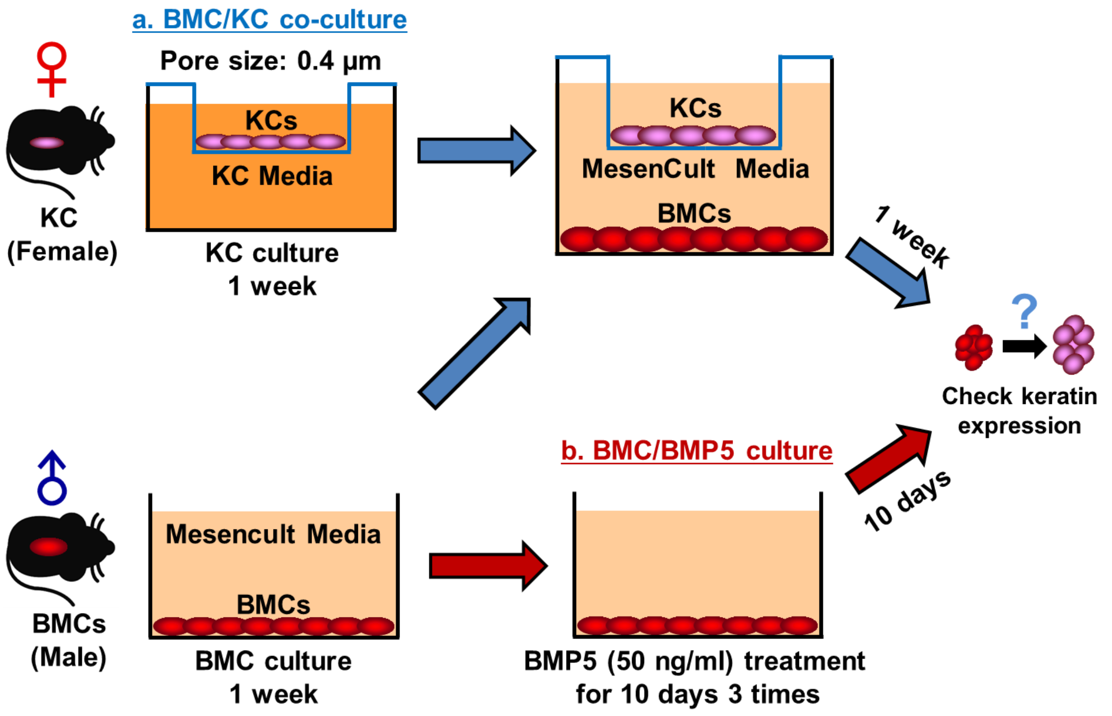


**Supplementary Figure 1. Overview of induction of keratin expressing cells in BMC culture:**

Plastic-adherent BMCs were co-cultured with KCs or subsequently were treated with BMP5. To perform (**a**) BMC/KC co-culture (blue arrows), KCs were harvested from dorsal skin of 7- to 9-week-old female C57BL/6 mice and cultured in KC medium on the membranes of inserts for five days until ~60% confluent, then switched to mouse MSC culture medium (MensenCult). BMCs were isolated from 7- to 9-week-old male C57BL/6 mice by flushing femurs and tibiae, and seeded on 6-well plates in the presence of MesenCult medium. Non-adherent cells were removed 48 hours after cell seeding, and medium was changed every three days. One week after KC harvest, adherent BMC culture (6-well plate) and KC culture (insert) were combined in the presence of MesenCult medium. One week after combining, co-cultured BMCs were collected to determine keratin expression from the cells. To perform (**b**) BMP5 treatment (red arrows), BMCs were isolated from male C57BL/6 mice as previously described. One week after culture, BMP5 (50 ng/ml) was added immediately after changing the medium. BMP5 was administered every three days for ten days. BMP5-treated BMCs were collected to determine keratin expression. *Some images in this figure were generated with AutoDraw.

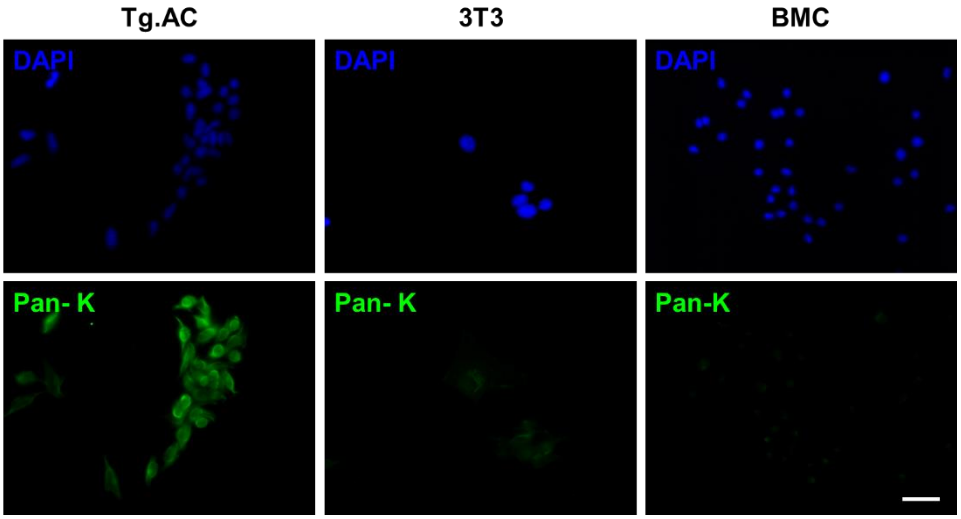


**Supplementary Figure 2. Immunostaining for Pan-keratin-positive and negative control, and plastic-adherent BMCs without treatment.**

IF staining controls for Fig. 1c-e. Tg.AC is a KC cancer cell-line used as keratin-positive control. Swiss mouse 3T3 is a fibroblastoid cell-line used as keratin-negative control. Primary BMCs were harvested and cultured without treatment as treatment-negative control. BMC harvest and culture occurred at the same time as the experimental group and are uniformly negative for keratin immunoreactivity. *White scale bar, 50 µm.

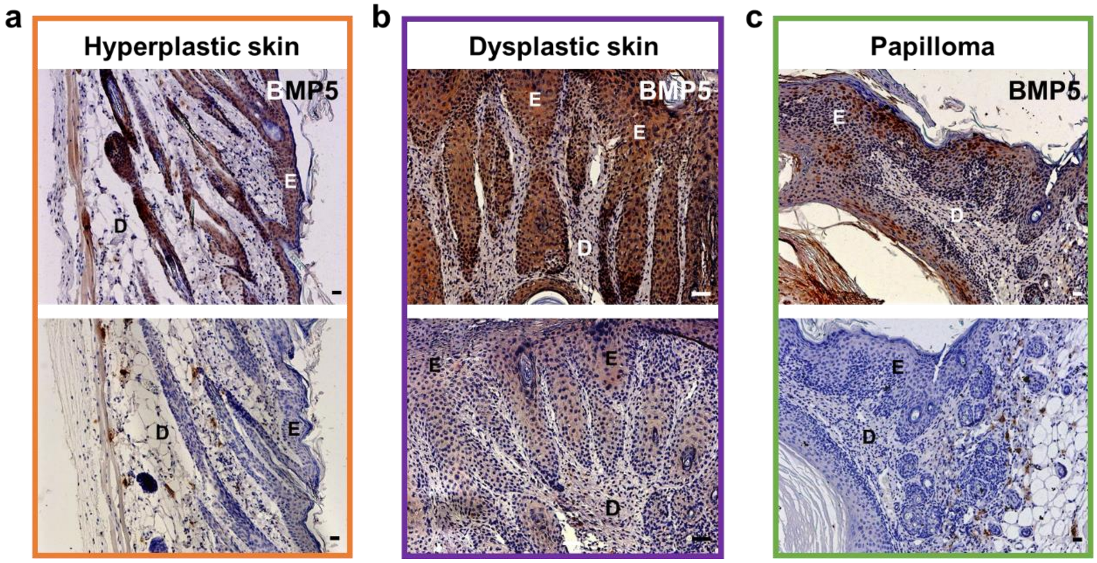


**Supplementary Figure 3. BMP5 expression in epithelium of (a) hyperplastic skin, (b) dysplastic skin, and (c) papilloma**.

BMP5 expression is detected from epithelium of all three samples (upper panels) and no signal is detected without primary BMP5 antibody (lower panels).
*Black and white scale bar, 50 µm.

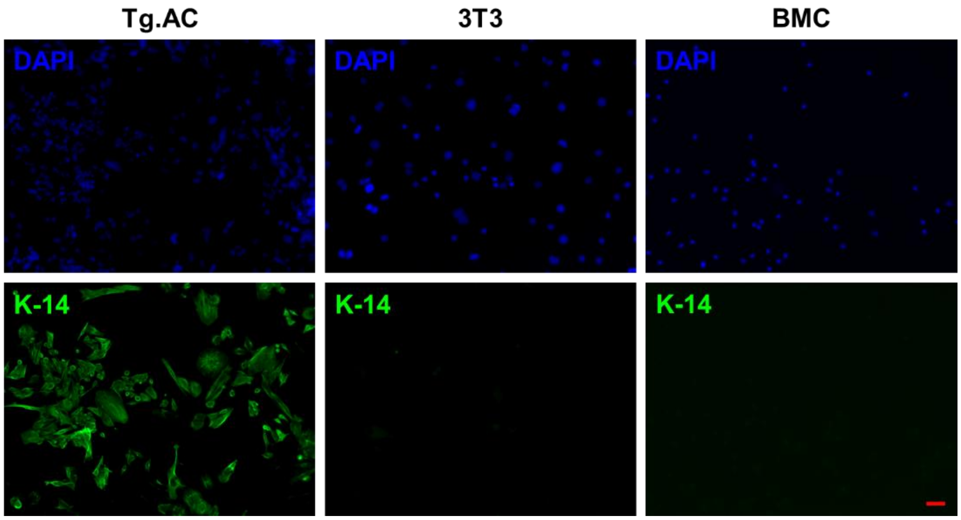


**Supplementary Figure 4. Immunostaining for K14-positive and negative controls and plastic-adherent BMCs without treatment.**

IF staining controls for Fig. 1f. Tg.AC is a KC cancer cell-line used as keratin-positive control. Swiss mouse 3T3 is a fibroblastoid cell-line used as keratin-negative control. Primary BMCs were harvested and cultured without treatment as treatment control. BMC harvest and culture occurred at the same time as the experimental group and are uniformly negative for keratin immunoreactivity. *Red scale bar, 200 µm.

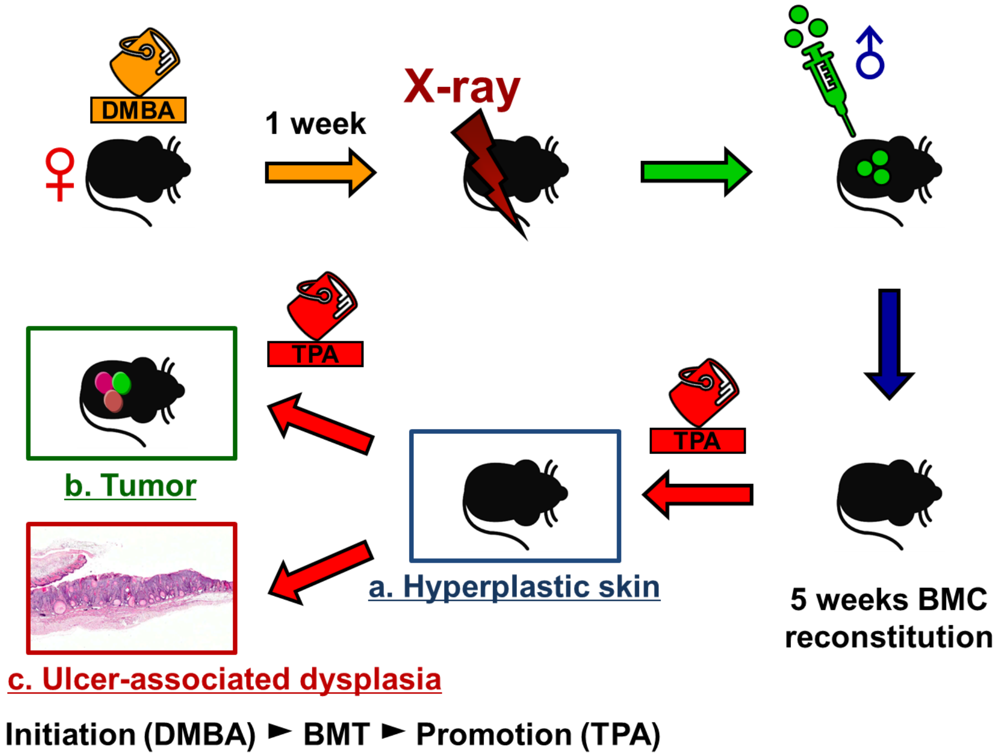


**Supplementary Figure 5. Overview of treatments with C57BL/6 recipients:**

To demonstrate recruitment of BMDCs into the TPA-treated epithelium of the skin and tumors, DMBA initiation was performed before gender-mismatched BMT and TPA promotion, which followed 5 weeks after BMT (DMBA ► BMT ► TPA). The dorsal fur of C57BL/6 female BMT recipient mice was clipped with electric clippers. One week after removing the dorsal fur, the mice received a single topical application of the carcinogen DMBA (200 nmol in 200 µl of acetone). One week after DMBA treatment, gender-mismatched BMT was performed with prepared mice. GFP-positive whole BMCs were collected from male donors and were transplanted to lethally irradiated female mice. 5 weeks after BMT (6 weeks after subtumorigenic carcinogen exposure), the mice received thrice-weekly treatments with the tumor promoter TPA (17 nmol in 200 µl of acetone). (**a**) Hyperplastic skin samples were collected from euthanized mice at different intervals during promotion (4 and 9 weeks, or 0, 5, and 15+ weeks). (**b**) Papilloma and (**c**) ulcer-associated dysplasia samples were obtained for further experiments. The following groups were prepared: No BMT (normal C57BL/6) with DMBA/TPA treatment control group, BMT without treatment (no DMBA/TPA) control group, BMT with DMBA/acetone control group, and BMT with DMBA/TPA experimental group. *Some images in this figure were generated with AutoDraw.

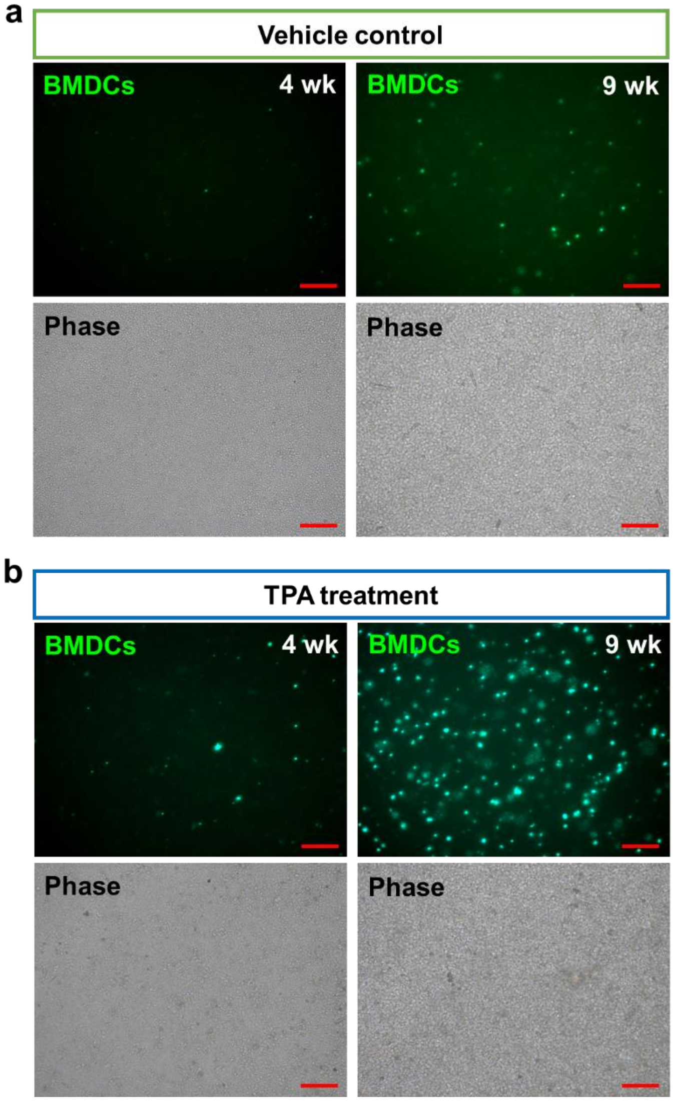


**Supplementary Figure 6. Long-term TPA treatment increased the number of BMDCs in the skin.**

GFP-expressing BMDCs are directly detected from primary KC harvest from (**a**) vehicle (acetone) and (**b**) TPA-treated dorsal skin of recipients after 4 and 9 weeks of treatment. *Red scale bar, 200 µm.

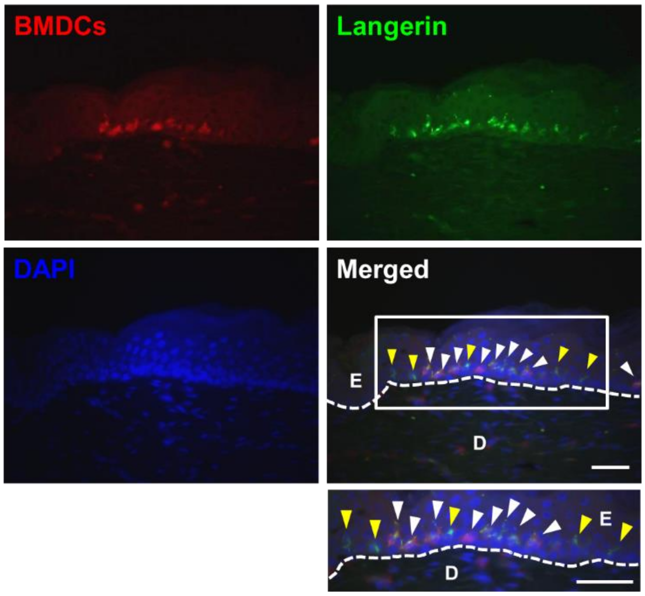


**Supplementary Figure 7. BM-derived GFP/langerin-positive cells located in the epidermis.**

A group of GFP/langerin-positive (double positive) cells (white arrowheads) and langerin-positive (single positive) cells (yellow arrowheads) were identified in the hyperplastic skin. White box area is magnified. *White scale bar, 50 µm.

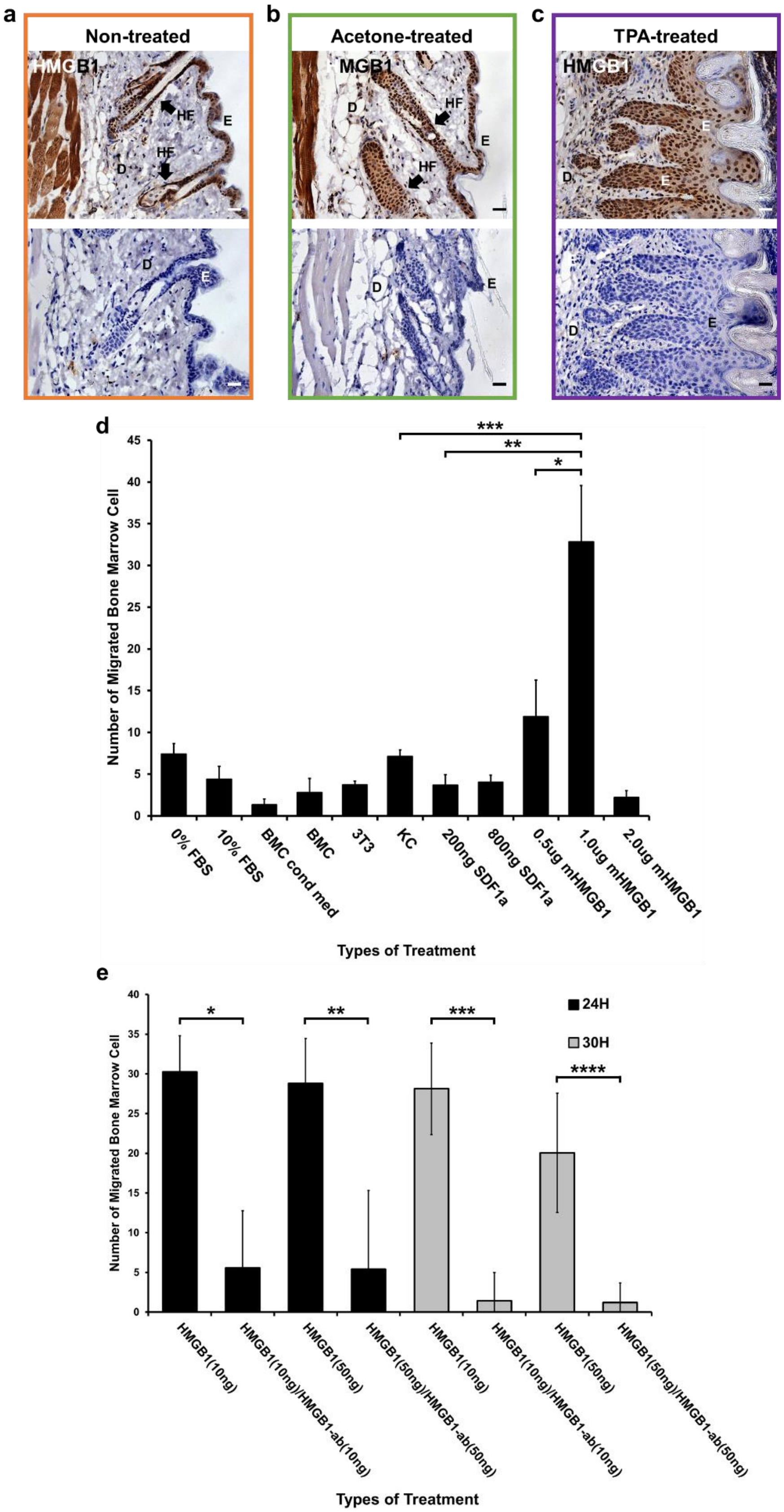
a
Non-treated
HMGB1
HF
E
D
b
Acetone-treated
MGB1
c
TPA-treated
HMGB1
d
Number of Migrated Bone Marrow Cell
0% FBS
10% FBS
BMC cond med
BMC
3T3
KC
200ng SDF1a
800ng SDF1a
0.5ug mHMGB1
1.0ug mHMGB1
2.0ug mHMGB1
Types of Treatment
e
24H
30H
HMGB1(10ng)
HMGB1(10ng)/HMGB1-ab(10ng)
HMGB1(50ng)
HMGB1(50ng)/HMGB1-ab(50ng)
Types of Treatment

**Supplementary Figure 8. Epidermal KCs and HMGB1 promote migration of BMCs.**

**a-c** (**a**) non-treated, (**b**) acetone-treated, and (**c**) TPA-treated mouse skin paraffin sections were prepared for IHC staining. HMGB1 expression is detected from epithelium of all three samples (upper panels) and no signal is detected without primary HMGB1 antibody (lower panels). Note the increased stromal reactivity in the TPA-treated groups. **d** Chemotaxis cell migration assay was performed with various conditions including negative and positive controls. Epidermal KCs, Swiss mouse 3T3 cells, HMGB1, and SDF1a were used as bait, epidermal KCs used as a positive control, and medium without serum or growth factors used as negative controls (n = 3, triplicate wells, *P = 7.57 × 10E-7, **P = 1.21 × 10E-7, ***P = 4.09 × 10E-7, P values are determined by Student's *t*-test, mean ± s.d.). Note that a significant number of BMCs migrated towards the epidermal KC and HMGB1 bait groups. In contrast, the SDF1a groups are less effective as bait. **e** HMGB1 antibody treatment blocked BMC migration in the presence of HMGB1. 10 and 50 ng of HMGB1 antibody were added in the presence of equivalent amounts of HMGB1 and incubated for 24 and 30 hours. The number of migrated BMCs was significantly decreased in HMGB1 antibody-treated groups but not in HMGB1-treated control groups without antibody (n = 3, triplicate wells, *P = 1.68 × 10E-7, **P = 6.1 × 10E-7, ***P = 2.63 × 10E-7, ****P = 1.5 × 10E-7, P values are determined by Student's *t*-test, mean ± s.d.). *Black and white scale bar, 50 µm.

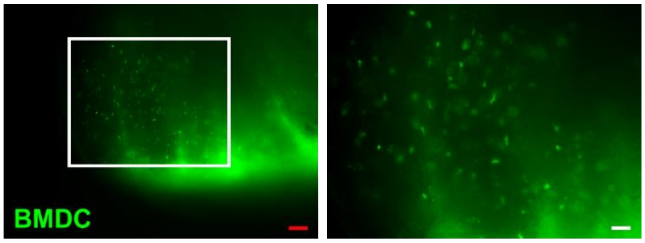


**Supplementary Figure 9. EGFP-expressing BMDCs are directly observed in the bisected whole mounted tumor.**

White box area is magnified. *Red scale bar, 200 µm; White scale bar, 50 µm.

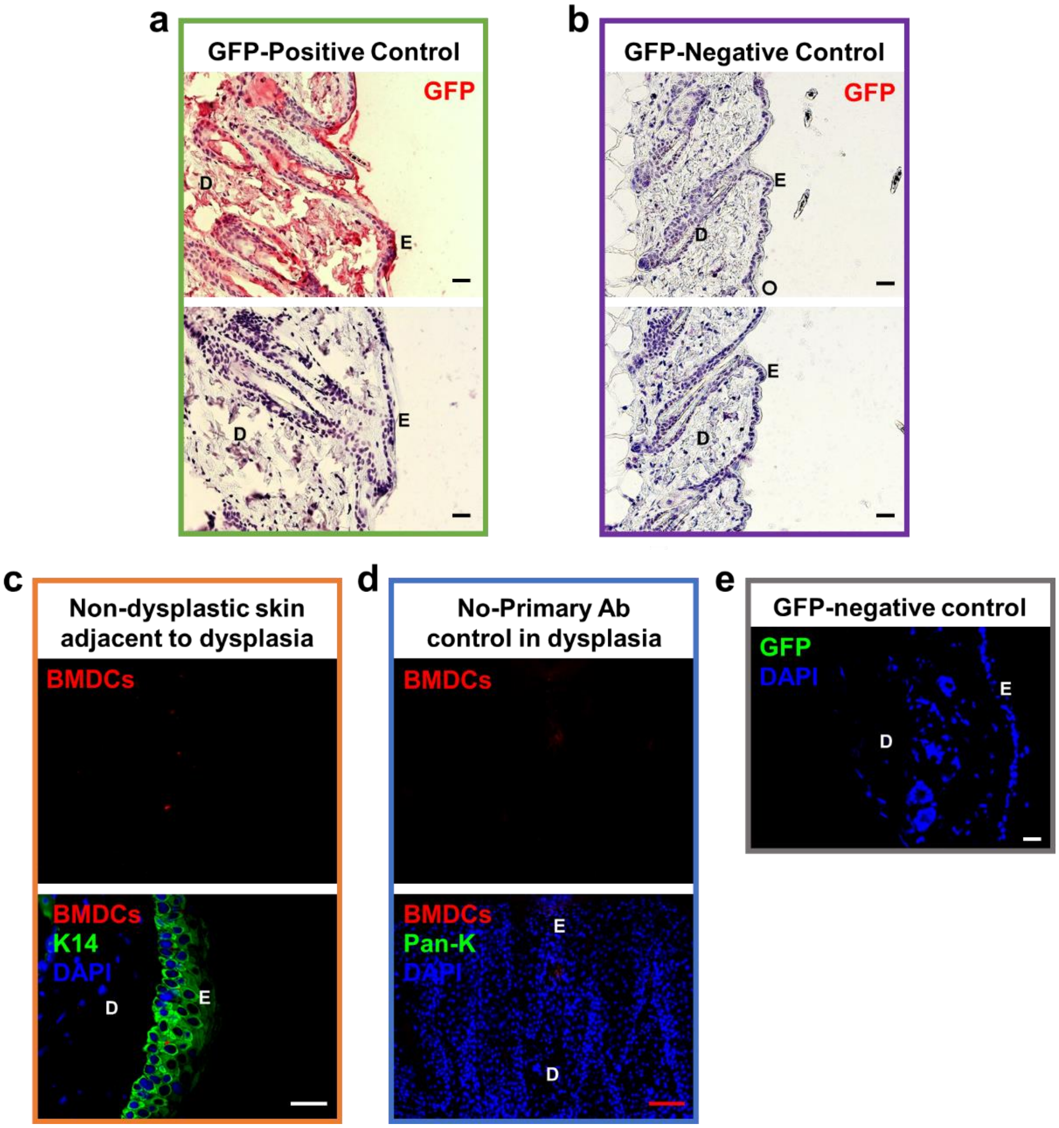


**Supplementary Figure 10.**

**a, b** Immunohistochemistry staining for (**a**) GFP-positive and (**b**) negative controls. GPF-positive (red) signal is only detected from positive control (upper panel of a) and no signal is detected from GFP-negative control (upper panel of b) in the presence of GFP antibody and without primary GFP antibody controls (lower panel of a and b). GFP-positive control skin prepared from EGFP mouse and negative control skin prepared from C57BL/6 mouse. GFP IHC controls for Fig. 3a, b, f, g, i, Fig. 4b, c. **c** BMDCs are not detected in non-dysplastic epidermis. **d** Positive signal is not detected in dysplasia without primary antibody (staining-negative control of Fig. 4d, e, g) **e** A micrograph of a frozen section of skin is from GFP-negative C57BL/6 mouse (GFP-negative control of Fig. 4h). *Black and white scale bar, 50 µm; red scale bar, 200 µm.

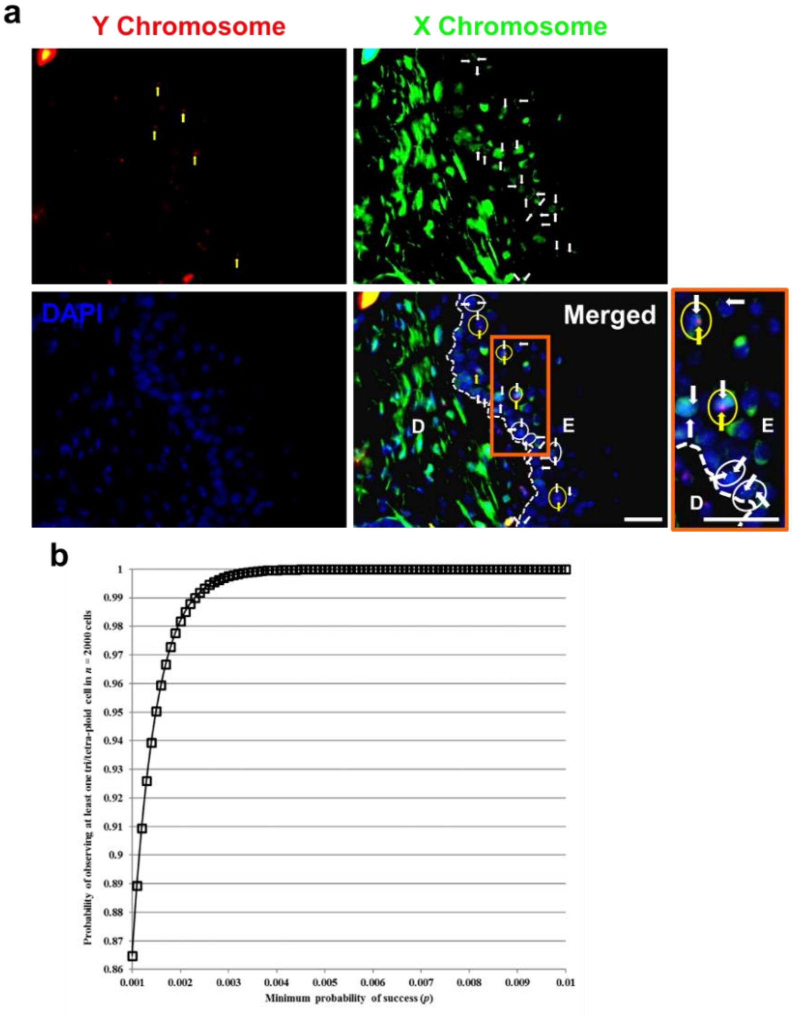


**Supplementary Figure 11. Evidence of cell fusion could not be detected by XY-FISH.**

**a** XY-FISH was performed with dysplastic samples and XXXY- or XXXX-positive cells are not detected. Only XX- or XY- positive cells are detected in the epidermis of samples. Yellow arrows indicate Y chromosomes. White arrows indicate X chromosomes. Yellow circles indicate XY-positive cells, White circles indicate XX-positive cells in the epidermis. Orange box area is magnified. **b** Probability of observing at least one tri/tetraploid cell in a set of 2,000 observed cells over a range of probabilities $p$. X and Y chromosome signals were counted in tissue samples after XY-FISH. The probability of observing at least one tri/tetraploid cell in 2000 was computed using Formula (3). The probability results for values of $p$ over the range $0.001 \leq p \leq 0.010$ in steps of 0.0001. The result of $k$ = 0 suggests that the true probability of observing a tri/tetraploid cell in a single experiment is small. In addition, the 99% confidence interval of the true probability of the probability $p$ was computed using the method implemented in the BINOM program. *White scale bar, 50 μm.

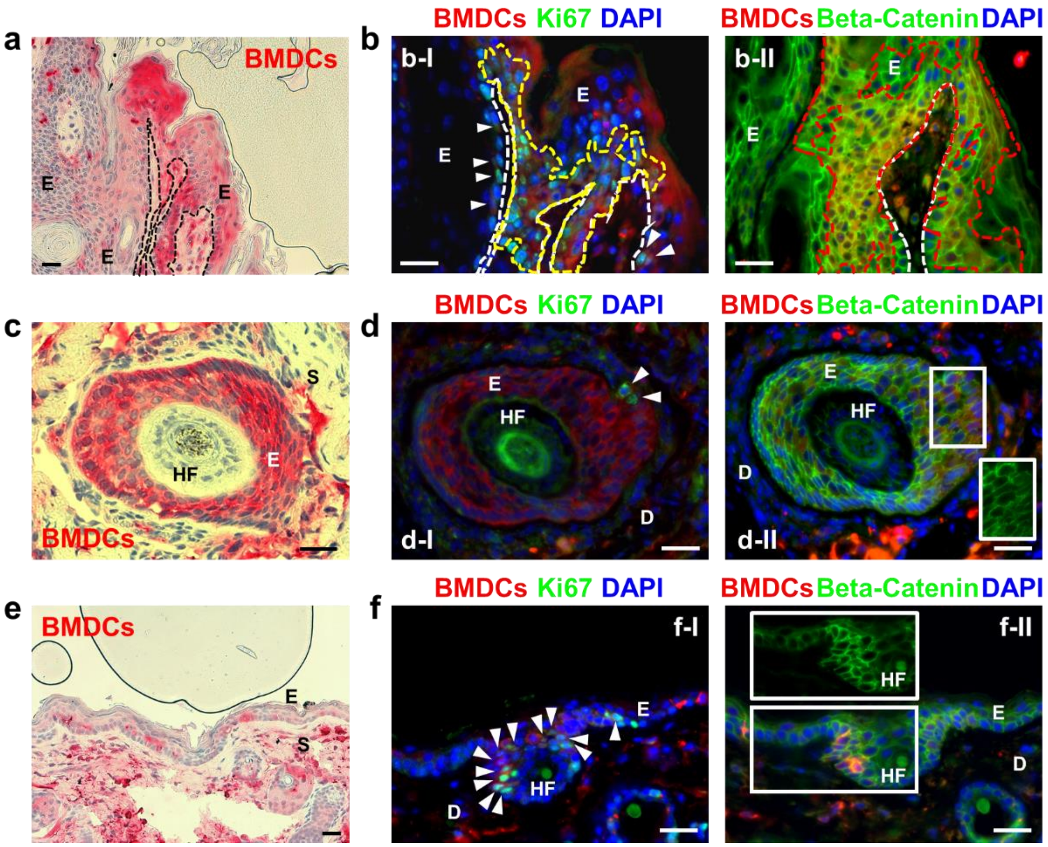


**Supplementary Figure 12. Staining of serial sections of tumor and skin samples with GFP, Ki67 and Beta-catenin.**

**a, b** Serial section staining of the papilloma sample with GFP (IHC and IF; a, b), Ki67 (IF; b-I) and Beta-catenin (IF; b-II). **c, d** Serial section staining of the papilloma sample with GFP (IHC and IF; c, d), Ki67 (IF; d-I) and Beta-catenin (IF; d-II). **e, f** Serial section staining of tumor-adjacent hyperplastic epidermis sample with GFP (IHC and IF; e, f), Ki67 (IF; f-I) and Beta-catenin (IF; f-II). Detailed description of picture is in figure legend of Fig. 3 and Fig. 5. *Black and white scale bar, 50 µm.

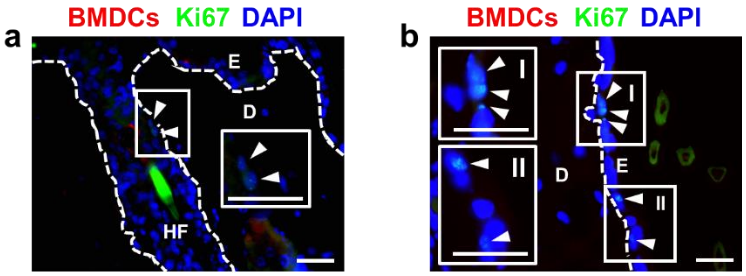


**Supplementary Figure 13. Ki67-positive cells in normal mouse skin (epidermis and hair follicle).**

**a, b** Paraffin skin sections from C57BL/6 mouse were prepared for GFP-negative control and Ki67 expression was determined in normal mouse skin as well. Small number of Ki67-positive cells are identified in normal skin. (**a**) Ki67-positive cells are located around HF bulge area (white arrowheads, white box area is magnified). (**b**) Ki67-positive cells in interfollicular epidermis (white arrowheads, area of white box-I and II are magnified). Around three Ki67-positive cells are observed between HFs in normal interfollicular epidermis. *White scale bar, 50 µm.

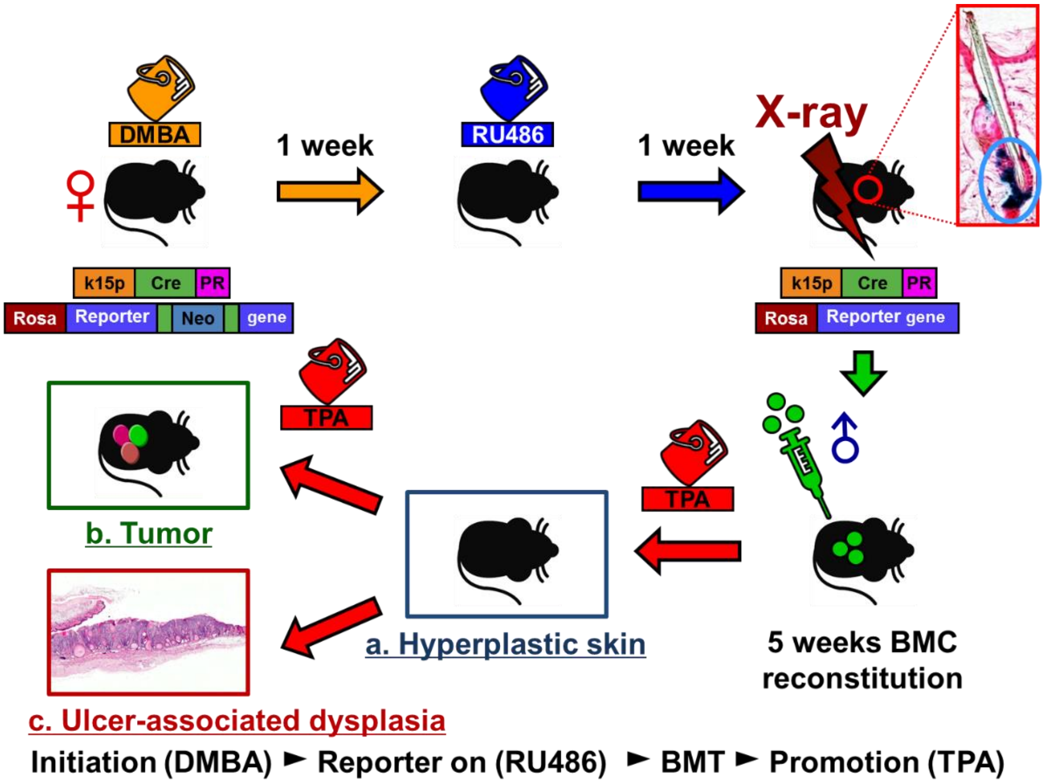


**Supplementary Figure 14. Overview of treatments with *Krt1-15CrePR1;R26R* recipients:**

To demonstrate contribution of K15-expressing cells and BMDCs in the hyperplastic epidermis, papillomas and ulcer-associated dysplasia, DMBA initiation and RU486 treatment were performed with female *Krt1-15CrePR1;R26R* mice before gender-mismatched BMT with TPA promotion, which followed 5 weeks after BMT (DMBA ► RU486 ► BMT ► TPA). The dorsal fur of BMT female recipient mice was clipped with electric clippers. One week later, the mice received a single topical application of the carcinogen DMBA (200 nmol in 200 µl of acetone). One week later, *Cre* recombinase was activated following daily topical applications of RU486 (1 mg in Neutrogena Hand Cream) for a total of 5 days. One week after RU486 treatment, gender-mismatched BMT was performed with prepared mice. GFP-positive whole BMCs were collected from male donors and were transplanted to lethally irradiated female mice. 5 weeks after BMT (7 weeks after subtumorigenic carcinogen exposure), the mice received thrice-weekly treatments with the tumor promoter TPA (17 nmol in 200 µl of acetone). (**a**) Hyperplastic skin, (**b**) papilloma, and (**c**) ulcer-associated dysplasia samples were collected from euthanized mice. *Some images in this figure were generated with AutoDraw.

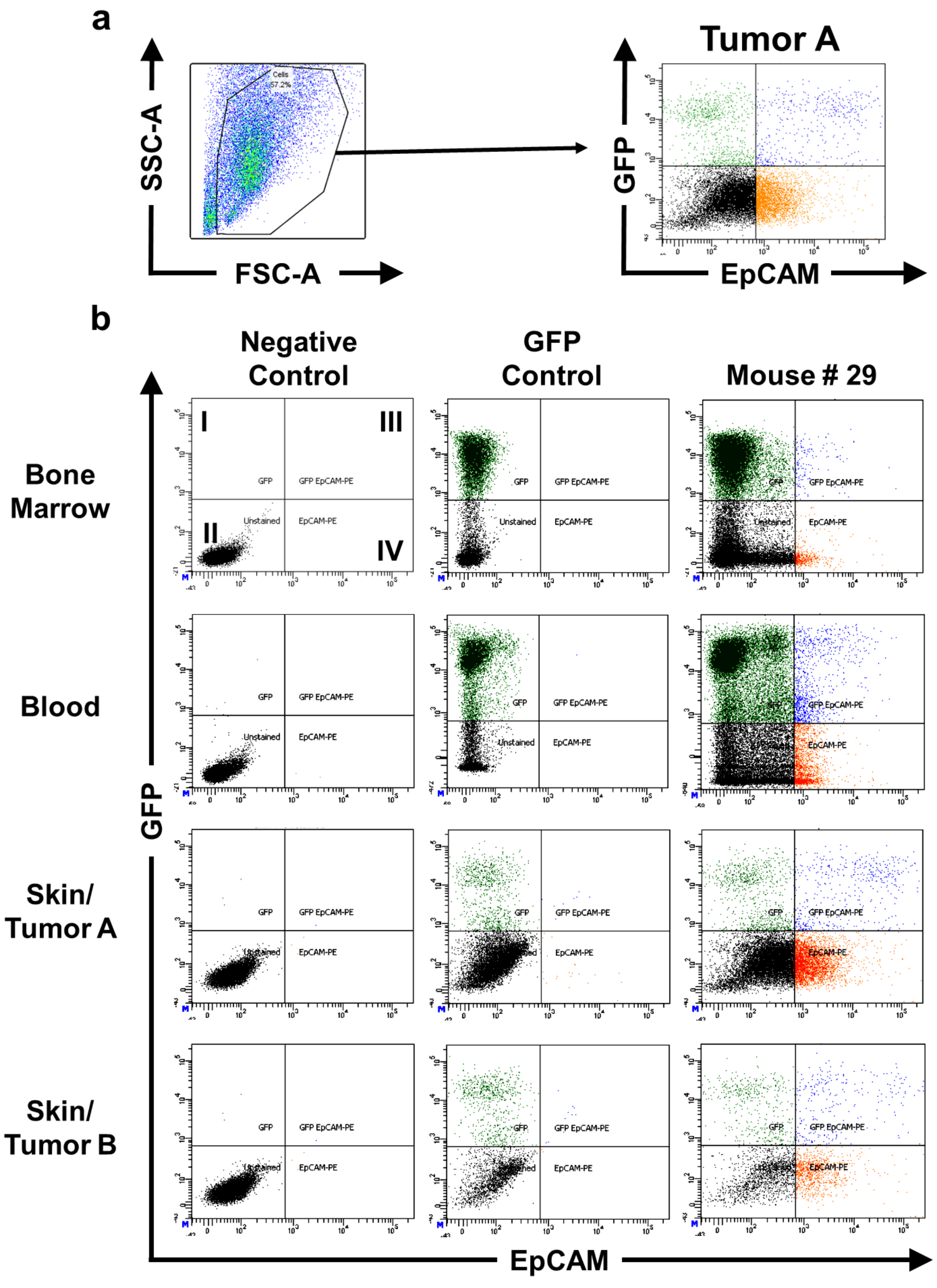
a
SSC-A
FSC-A
Cells
57.2%
Tumor A
GFP
EpCAM
b
Negative Control
GFP Control
Mouse # 29
Bone Marrow
Blood
Skin/ Tumor A
Skin/ Tumor B
I
II
III
IV
GFP
GFP EpCAM-PE
Unstained
EpCAM-PE
GFP
EpCAM

**Supplementary Figure 15. Gating strategy and positive/negative controls for Fig. 7c flow cytometry analysis:**

**a** Prior to GFP versus EpCAM analysis, samples were gated using forward versus side scatter. **b** Blood, BM, and skin and/or two disaggregated squamous tumor samples were collected from FVB/N (GFP-negative control), FVB/EGFP (GFP-positive control) and DMBA-exposed BMT recipient (#29, FVB/N). Expression level of GFP (cells from FVB/EGFP donor, Y-axis) and EpCAM (epithelial cell marker, X-axis) were determined by FACS. *quadrant I = $GFP^+/EpCAM^-$, quadrant II = $GFP^-/EpCAM^-$, quadrant III = $GFP^+/EpCAM^+$, quadrant IV = $GFP^-/EpCAM^+$.
